# Adeno-associated virus as a delivery vector for gene therapy of human diseases

**DOI:** 10.1038/s41392-024-01780-w

**Published:** 2024-04-03

**Authors:** Jiang-Hui Wang, Dominic J. Gessler, Wei Zhan, Thomas L. Gallagher, Guangping Gao

**Affiliations:** 1 Horae Gene Therapy Center, University of Massachusetts Chan Medical School, Worcester, MA 01605 USA; 2 Department of Microbiology and Physiological Systems, University of Massachusetts Chan Medical School, Worcester, MA 01605 USA; 3 Centre for Eye Research Australia, Royal Victorian Eye and Ear Hospital, East Melbourne, VIC 3002 Australia; 4 Ophthalmology, Department of Surgery, University of Melbourne, East Melbourne, VIC 3002 Australia; 5 Department of Neurological Surgery, University of Massachusetts Chan Medical School, Worcester, MA 01605 USA; 6 Department of Neurosurgery, University of Minnesota, Minneapolis, MN 55455 USA; 7 Department of Medicine, University of Massachusetts Chan Medical School, Worcester, MA 01605 USA; 8 Li Weibo Institute for Rare Diseases Research, University of Massachusetts Chan Medical School, Worcester, MA 01605 USA

**Keywords:** Drug delivery, Molecular biology

## Abstract

Adeno-associated virus (AAV) has emerged as a pivotal delivery tool in clinical gene therapy owing to its minimal pathogenicity and ability to establish long-term gene expression in different tissues. Recombinant AAV (rAAV) has been engineered for enhanced specificity and developed as a tool for treating various diseases. However, as rAAV is being more widely used as a therapy, the increased demand has created challenges for the existing manufacturing methods. Seven rAAV-based gene therapy products have received regulatory approval, but there continue to be concerns about safely using high-dose viral therapies in humans, including immune responses and adverse effects such as genotoxicity, hepatotoxicity, thrombotic microangiopathy, and neurotoxicity. In this review, we explore AAV biology with an emphasis on current vector engineering strategies and manufacturing technologies. We discuss how rAAVs are being employed in ongoing clinical trials for ocular, neurological, metabolic, hematological, neuromuscular, and cardiovascular diseases as well as cancers. We outline immune responses triggered by rAAV, address associated side effects, and discuss strategies to mitigate these reactions. We hope that discussing recent advancements and current challenges in the field will be a helpful guide for researchers and clinicians navigating the ever-evolving landscape of rAAV-based gene therapy.

## Introduction

**Fig. 1:**
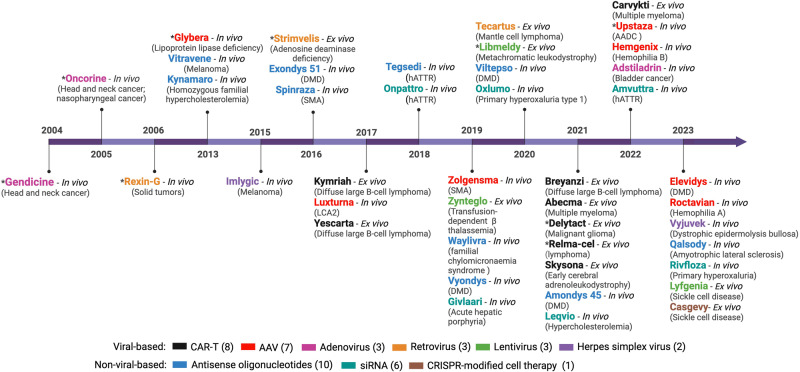
Approved gene therapy products and delivery platforms. Gene therapy products are generally developed for 1) Ex vivo gene therapy where affected patient cells are isolated, genetically modified in cell culture, expanded, enriched, and reinfused into patients to function as a living drug (e.g., CAR-T cells, *Lyfgenia*, *Casgevy*) and 2) In vivo gene therapy which is administered directly to patients to achieve therapeutic effects (e.g., *Gendicine*, *Kynamaro*, *Imlygic*, *Luxturna*, *Onpattro*). Delivery platforms for gene therapy drugs are primarily categorized into two groups: viral and non-viral-based. Viral-based gene therapy utilizes viruses as gene delivery vectors, including AAV, adenovirus, retrovirus, lentivirus, and herpes simplex virus. Non-viral-based gene therapy includes antisense oligonucleotides, siRNAs, and cell-based CRISPR genome editing. CAR-T chimeric antigen receptor (CAR) T cell therapy, SMA spinal muscular atrophy, AADC aromatic L-amino acid decarboxylase deficiency DMD Duchenne muscular dystrophy, LCA2 Leber congenital amaurosis type 2, hATTR hereditary transthyretin amyloidosis. * indicates non-FDA-approved gene therapy. Figure created with Biorender.com

Gene therapy represents a groundbreaking approach for addressing genetic diseases, employing a range of strategies to modify gene expression within target cells using non-viral or viral vehicles.^1^ One common strategy is gene replacement therapy, where a functional copy of the faulty gene is introduced to living cells. A significant milestone for this strategy was reached in 2017 when the US Food and Drug Administration (FDA) approved the first gene therapy product, Luxturna, a gene replacement therapy for Leber congenital amaurosis type 2 (LCA2). Alternatively, gene silencing therapy aims to suppress or silence a target gene, mainly through RNA interference. For example, Patisiran, a small interfering RNA, is employed in the treatment of hereditary transthyretin amyloidosis.^2^ Another strategy is genome editing, which can be facilitated by clustered regularly interspaced short palindromic repeats (CRISPR)-based technologies to allow direct modifications of the somatic genome. Modified CRISPR systems that can perform RNA editing are also being developed. Moreover, gene expression may be altered epigenetically via DNA methylation, histone modification, and microRNA regulation. This method offers the advantages of being reversible and versatile, which allows it to be adaptable based on disease progression and responses to treatment. Innovative strategies such as leveraging suppressor tRNAs to enable readthrough of premature stop codons offer avenues to rescue pathologic nonsense mutations and restore gene function under endogenous regulation.^3^ Irrespective of the chosen strategy, gene therapy can be implemented either ex vivo or in vivo.^4^ Ex vivo gene therapy involves extracting patient cells, genetically modifying them, and reintroducing them back to the patients. In contrast, in vivo gene therapy directly delivers genetic materials to the target tissues. As of now, a total of 14 ex vivo and 29 in vivo gene therapies have obtained approval mainly through the FDA (Fig. 1). Of these approved gene therapies, 17 are non-viral-based, while 26 are viral-based. Various viral vectors have been studied for in vivo gene therapy, including adenovirus (Ad), retrovirus, lentivirus, and herpes simplex virus (HSV). Adeno-associated virus (AAV) vectors have emerged as the preferred choice in clinical trials and FDA-approved applications (Fig. 1). This is because of their broad tissue tropism, relatively good safety profile, and versatile manufacturing processes. Importantly, AAV is non-pathogenic, rarely integrates into the host genome, and can sustain long-term transgene expression. Moreover, vectors based on some AAV serotypes are inherently capable of efficient cellular entry and transgene expression, which enhances transduction efficacy.^5^

**Fig. 2:**
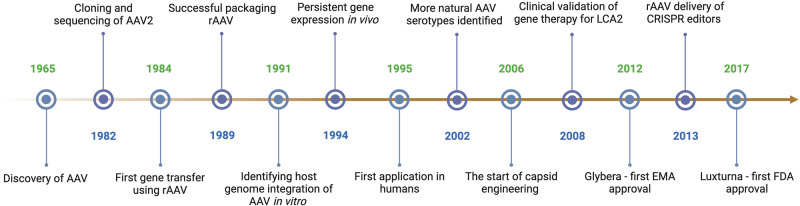
Historical milestones in AAV biology research and gene therapy development. Decades of studying AAV biology have led to crucial advances in understanding its structure, biology, vectorology, and gene therapy applications. Historical milestones in AAV research and development are summarized chronologically. These advancements paved the way for successful clinical trials and regulatory approvals for rAAV-based gene therapeutics to treat various human diseases. Figure created with Biorender.com

Originally discovered as a contaminant in Ad preparations in the mid-1960s,^6,7^ wild-type AAV (wtAAV) is a non-enveloped virus containing a single-stranded DNA (ssDNA) genome with inverted terminal repeats (ITRs) at both ends (Fig. 2). Studies of AAV biology led to the successful cloning and sequencing of the wtAAV2 genome.^8–11^ In the 1990s and 2000s, diverse AAV serotypes with distinct tissue tropisms were identified, thereby introducing avenues for targeted gene delivery approaches.^12,13^ Studies reported that the wtAAV genome can be integrated into the host chromosome, facilitating stable, long-term transgene expression.^14,15^ During the 2000s, work was done to engineer AAV capsids to enhance tissue specificity and transduction efficacy as well as to improve the safety of recombinant AAV (rAAV).^16^ This has advanced to successful rAAV-based clinical trials, particularly for monogenic rare diseases, resulting in regulatory approvals of rAAV gene therapy products.^17,18^ In parallel, various methods to scale up rAAV manufacturing have been developed, leading to growing interest in rAAV-based gene therapy. More recently, the advent of CRISPR technology has revolutionized the landscape of gene therapy, enabling precise gene editing delivered into cells via rAAV.^19^

rAAV-based gene therapy has emerged as a potential cure for once-untreatable genetic diseases owing to its distinctive attributes, including its small size, non-pathogenic nature, versatile tissue tropism, episomally durable transgene expression, replication incompetence, engineerable capsids, and adaptability for diverse payload delivery.^16,20^ Despite some early successes, numerous concerns and challenges have restrained its widespread applications, particularly for complex disorders. These include limited cargo capacity, immune responses to high systemic doses, potential genotoxicity, challenges in achieving tissue specificity, and manufacturing complexity.^16,20^ Here, we describe AAV biology and engineering that is the foundation of rAAV gene therapy, discuss key principles of AAV vectorology and the current manufacturing methodologies, and provide overviews on clinical applications and challenges of rAAV gene therapy for treating a wide range of human diseases. Finally, we identify gaps in our current understanding, propose potential solutions to ongoing challenges, and outline future directions in this rapidly advancing field.

## AAV biology and vector engineering

### AAV as a natural virus

#### Structure and genome

AAV has an icosahedral capsid composed of 60-mer subunits and a 4.7 kb ssDNA genome flanked by a 145 bp T-shaped ITR at each end. The capsid is assembled by viral proteins (VPs) VP1, VP2, and VP3 at a ratio of 1:1:10.^21^ VP3 is composed of conserved β-strands which are linked by surface-exposed variable loops. These structures shape the AAV surface morphology and determine AAV serotype-specific functions. The AAV capsid has a channel with a pore-like opening at each five-fold axis, protrusions surrounding the three-fold axis, and a depression at the two-fold axis.

The wtAAV genome contains two main open reading frames encoding four non-structural replication genes (*Rep*) and three structural capsid genes (*Cap*) along with an ORF for the assembly-activating protein, which is involved in capsid assembly.^22^ In addition, the function of membrane-associated accessory protein (MAAP), which is encoded on a different reading frame within the *Cap* gene, remains unclear.^23^ The ITRs largely serve as the viral origins of replication and provide the signal for packaging.^24^

#### Seroprevalence and non-pathogenic nature

Distinct serotypes of wtAAV are characterized by variations of VPs and ITRs and have been isolated primarily from humans and non-human primates (NHPs).^12,13,25^ Human seroprevalence studies have revealed that a significant proportion of populations possess neutralizing antibodies (NAbs) against multiple AAV serotypes. A study of 888 human serum samples from healthy volunteers found that NAbs against AAV2 were the most prevalent, followed by AAV1, AAV7, and AAV8.^26^ Another study showed that the most prevalent NAbs against serotypes other than AAV2 in serum from 552 human samples were AAV1 (74.9%), AAV6 (70.1%), AAV5 (63.9%), AAV8 (60.4%), and AAV9 (57.8%).^27^

As a *Dependoparvovirus* within the *Parvoviridae* family, wtAAV requires essential genes from a helper virus, such as Ad or HSV to facilitate the replication and transcription of its genome, including Ad *E1*, *E2a*, *E4*, and *VA RNA* genes.^28,29^ As a result, wtAAV cannot independently replicate and produce new viral particles but can establish a latent infection in host cells by persisting as episomal circular monomers or concatemers. Notably, in in vitro infections, approximately 0.1% of wtAAV2 genomes integrate into a specific region on the long arm of chromosome 19 (19q13-qter), termed the AAVS1 site.^30^ Although AAV is considered non-pathogenic, recent studies reported that AAV2 was connected with unexplained acute hepatitis in children worldwide.^31–33^ There is no direct evidence for the mechanism of how AAV2 triggers hepatitis, but it has been suggested that abnormal immune responses to AAV2 might mediate hepatoxicity.^34^ However, these studies acknowledged that COVID-19 infection may be a contributing factor and reported that most cases of acute hepatitis resolved without prolonged immunosuppression.

### rAAV as a delivery vector for gene therapy

#### Vectorology and tissue tropism

Naturally existing wtAAVs are rapidly evolving, generating a vast genomic diversity classified as viral “clades”. In the past two decades, at least 12 AAV serotypes and over 1000 variants have been identified from Ad stocks, human/NHP tissues, and other mammals or non-mammalian species.^5,12,13,25^ These serotypes have distinct preferences for various cells or tissues, which is known as tropism (Table 1).^35–39^ Genomic differences among AAV serotypes are primarily found in the variable regions of the virus capsid sequence, particularly VP3, which play a crucial role in determining the tropism. However, many other processes and interactions with host proteins may affect the tropism, including cell surface receptors, cellular intake, intracellular trafficking, nuclear import, viral uncoating, second-strand DNA synthesis, and genome circularization and concatemerization.

**Table 1:** Summary of natural AAV receptors and tissue specificity in humans

AAV serotype	Origin	Receptor for cellular attachment		Receptors for post-attachment	Tissue tropism in human
		Primary receptors	Co-receptors		
AAV1	NHP	N-linked sialic acid	Unknown	AAVR, GPR108	Skeletal muscle, CNS, airway, retina, pancreas
AAV2	Human	HSPG	FGFR1, HGFR, LamR, CD9, Tetraspanin	AAVR, GPR108	Retina, CNS
AAV3	Human	HSPG	FGFR1, HGFR, LamR	AAVR, GPR108	Liver
AAV4	NHP	O-linked sialic acid	Unknown	GPR108	Lung
AAV5	Human	N-linked sialic acid	PDGFR	AAVR	Retina, CNS, kidney, pancreas, liver
AAV6	Human	N-linked sialic acid	EGFR	AAVR, GPR108	Airway, CNS
AAV7	NHP	Unknown	Unknown	Unknown	Liver
AAV8	NHP	Unknown	LamR	AAVR, GPR108	Liver, CNS, retina
AAV9	Human	Galactose	LamR	AAVR, GPR108	Heart, CNS
rh8	NHP	Unknown	Unknown	Unknown	CNS
rh10	NHP	Unknown	Unknown	Unknown	CNS, skeletal muscle
rh74	NHP	Unknown	Unknown	Unknown	Skeletal muscle

*CNS* central nervous system, *FGFR1* fibroblast growth factor receptor 1, *HGFR* hepatocyte growth factor receptor, *PDGFR* platelet-derived growth factor receptor, *EGFR* epidermal growth factor receptor, *LamR* 37/67 kDa laminin receptor, *AAVR* adeno-associated virus receptor, *GPR108* G protein-coupled receptor 108

The wtAAV2 genome was initially cloned in the 1980s,^8,9,11^ serving as a template for rAAV. rAAV possesses an identical capsid sequence and structure to that of wtAAV, but lacks any wtAAV protein-coding sequences, instead incorporating therapeutic gene expression cassettes that are within 4.7 kb packaging capacity. The only viral elements in the rAAV genome are the ITRs.^16^ rAAV is primarily produced by trans-complementing *Rep*/*Cap* and Ad helper genes in transiently transfected HEK293 cells. rAAV genomes with AAV2 ITRs can be trans-encapsidated with capsids of different AAV serotypes to alter their transduction properties.

#### Transduction pathways of rAAVs

Despite an improved understanding of wtAAV biology, the mechanisms by which rAAV interacts with cellular surfaces and delivers transgenes into the nuclei of host cells remain poorly understood. A growing body of research suggests that the virus capsid initially adheres to the cell surface through primary receptors such as glycans, glycoconjugates, or sialic acid, followed by interactions with co-receptor proteins.^40^ For example, rAAV2 binds to heparan sulphate proteoglycan (HSPG), rAAV1, rAAV4, and rAAV5 primarily interact with sialic acid, and rAAV9 interacts with N-linked galactose.^41,42^ The distinct binding sites on the capsid are thought to determine its tropism, therefore efforts are being made around these sites to engineer AAV variants with enhanced transduction capabilities for specific cells or tissues.^20^ Recent genome-wide screening studies identified novel host proteins facilitating rAAV transduction, including the type I transmembrane protein KIAA0319L, which has been designated as the AAV receptor (AAVR).^43^ Another universal host protein, G protein-coupled receptor 108 (GPR108), plays a role in the transduction of several rAAV serotypes.^39^ Although knocking out *AAVR* and *GPR108* in vivo reduces transduction, cell-surface binding is largely unaffected,^39,44^ suggesting that these host proteins primarily contribute to the transduction process post-attachment.

After binding to the cell surface, rAAV is internalized via endocytosis using different mechanisms, which can vary by cell types and rAAV serotypes.^40,45,46^ rAAV particles inside endosomes are subjected to pH-dependent conformational changes and then trafficked through the trans-Golgi network.^47,48^ The rAAV particles escape from the endosomes and trans-Golgi network and then enter the nucleus through the nuclear pore complex.^49,50^ Once the viral particles are inside the nucleus, the ssDNA genome is released and converted into double-stranded DNA (dsDNA) via a process known as second-strand synthesis. Transcription is then initiated from the self-primed ITR at the 3′ end of the genome.^51^ The genome can be made into a dsDNA structure by mutating an internal ITR motif, which allows for faster replication and enhanced transduction compared to the single-stranded rAAV (ssAAV) genome.^52^ However, this self-complementary AAV (scAAV) genome design reduces the packaging capacity by half.^53,54^ The dsDNA genome subsequently undergoes circularization and concatemerization, stabilizing the vector genome for episomal persistence in postmitotic cells.^16^ Notably, the ITR sequence can also serve as a recombinogenic element to facilitate vector genome recombination.^16^ The rAAV transduction pathway involves multiple cellular events and may fail or be destroyed by the host at any step, weakening or preventing transduction. Thus, a full understanding of this pathway will help identify additional key host factors affecting the transduction efficacy of rAAV.

### rAAV engineering

Both the rAAV capsid sequence and the genomic DNA cargo, including promoters, transgenes, enhancers, and ITRs, are under intense investigation. This section introduces several engineering strategies to modify rAAV for better transduction efficiency and tissue specificity.

#### Capsid engineering for transgene delivery

**Fig. 3:**
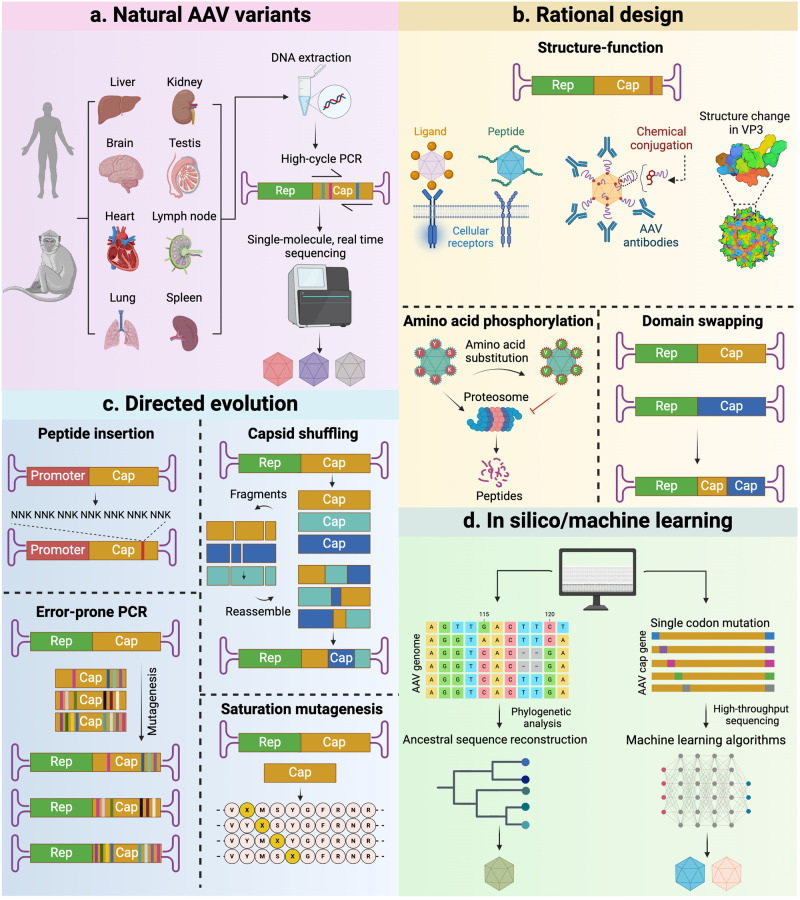
Strategies applied for the development of novel AAV variants. **a** Natural occurring AAV variants can be isolated from human and NHP tissues using high-cycle PCR and high-throughput sequencing. **b** Rational design utilizes knowledge of AAV biology to modify the relevant amino acids in AAV capsid to enhance the transduction capability or evade immune surveillance. **c** Directed evolution is an engineering approach to develop novel AAV variants with designated specificity, including random or defined peptide insertion, capsid shuffling, error-prone PCR, and saturation mutagenesis. **d** In silico approach utilizes known capsid sequences to reconstruct ancestral AAV sequences. Machine learning is being used for predicting the relationship between specific sequences in the AAV genome with packaging capabilities and tissue tropism by using large datasets of samples transduced with AAVs whose genome had been mutagenized. Figure created with Biorender.com

Capsid engineering is a principal strategy for developing rAAV tailored for different clinical applications. It mainly consists of three approaches: isolation of naturally occurring AAV variants, rational design, and directed evolution (Fig. 3). Recently, computer-based approaches using bioinformatic prediction and machine learning (ML) have emerged as novel tools to assist capsid engineering^23,55^ (Fig. 3).

##### Naturally occurring AAV variants

Early in AAV development, human tissues were crucial sources for discovering new AAVs. From 1965 to 2004, only a limited number of AAVs were derived primarily from human clinical samples, which were subsequently vectorized and subjected to testing.^5^ AAV9 was first identified in human liver tissue, yet it has strong brain tropism due to its ability to cross the blood-brain barrier (BBB), making it a common choice for central nervous system (CNS) transduction.^13^ AAV7 and AAV8 were isolated from the heart and lymph nodes of rhesus monkeys and demonstrated superb tropism in skeletal muscle and liver.^12^

Studies indicate that 40–80% of people have antibodies against known wtAAV serotypes, highlighting the necessity of discovering more sero-distinctive AAVs.^26,56^ Screening for AAV serotypes in species other than humans and NHPs has been done to find capsids that can avoid the neutralizing effects of encountering pre-existing antibodies against human AAVs,^57–60^ but these may have limited transduction in humans. Thus, discovering human-derived AAV variants and screening for candidates with enhanced transduction and specific tissue tropism has been a common approach. AAVv66, an AAV2 variant isolated from a human sample by long-read sequencing, exhibits enhanced production and CNS transduction compared to AAV2.^61^ These findings indicate that natural variants can provide valuable tools for improved tissue-specific transduction. While only a few groups continue to evaluate natural capsid variants as potential vectors for gene therapy, rAAVs derived from natural AAVs remain the dominant choice in current clinical studies.

##### Rational design

Rational design approaches attempt to make structural modifications in specific sites on rAAV capsids based on the understanding of rAAV structure and biology. Three key approaches have generally been employed: genetic mutation, insertion of nonviral domains to modify tissue affinity, and chemical modification.

Studies exploring the contributions of specific amino acid residues on rAAV transduction efficacy revealed that phosphorylation of surface tyrosine residues on rAAV2 capsids led to their degradation before nuclear entry.^62–65^ Modifying these residues to phenylalanine (Y444F/Y500F/Y730F) increased gene delivery in the CNS.^66^ While rAAV2 is not able to transduce photoreceptors through intravitreal injection, a variant containing substitutions of specific residues (Y272F/Y444F/Y500F/Y730F/T491V) achieved transduction of up to 25% of photoreceptor cells. Moreover, altering surface-exposed residues may enable tissue detargeting, thus improving the safety profile. For example, introducing H527Y and R533S substitutions, which were identified in samples from chimpanzee tissues, into the rAAV9 capsid resulted in reduced transduction in peripheral tissues after intravascular injection into both neonatal mice and a mouse model of a fatal pediatric leukodystrophy.^67^

Another rational design approach is to introduce functional domains into specific sites of the rAAV capsid. A study inserted a 15-mer binding domain of the human luteinizing hormone receptor (LH-R), resulting in the successful transduction in ovarian cancer cells in an HSPG-independent manner via the LH-R.^68^ By inserting cell-penetrating peptides into the rAAV9 capsid, a study identified two variants that were able to cross the BBB with improved CNS transduction after systemic delivery.^69^ The host immune response is a major barrier to rAAV being able to induce effective and long-term transgene expression. Structural studies have demonstrated that the NAbs recognition sites on rAAV capsids might be conserved.^70^ Engineered rAAV variants were able to evade NAbs in mice, NHPs, and human sera as determined by structural information from cryogenic electron microscopy (cryo-EM) images of rAAV1 capsids complexed with three murine monoclonal antibodies.^70^ Conjugating the rAAV capsid with biotin-polyethylene glycol (PEG)^71^ and N-acetylgalactosamine (GalNAc)^72^ may help evade NAbs, although this is potentially at the cost of altered tissue tropism and reduced transduction efficiency. One study used domain swapping to generate 27 chimeric *Cap* from rAAV2 and rAAV8 and demonstrated that the liver tropism achieved by rAAV8 was associated with the loop IV domain.^73^ Additionally, inserting arginine-glycine-aspartic acid (RGD) integrin binding motifs in AAVrh10’s variable region VIII improved cardiac-specific transduction and reduced liver distribution.^74^

Chemical modification without altering the amino acid composition of the rAAV capsid offers a promising approach in capsid engineering. A minor modification to the surface amino acids can alter the receptor binding affinity to affect transduction and tropism. A study used PEG-N-hydroxysuccinimide with an electrophilic succinimidyl propionic acid functional group to cross-link with lysine residues on rAAV2 capsid to enable NAb evasion.^75^ Glycated rAAV2 was found to have reduced binding to heparin and monoclonal antibody A20, resulting in remarkably improved transgene expression in muscles.^76^ One study covalently conjugated antibodies against skeletal muscle-specific protein CACNG1 to a variable region of rAAV9 capsid, leading to engineered capsids that can specifically express transgene in mouse myotubes with reduced liver targeting compared to unconjugated rAAV9.^77^ Likewise, another study engineered a hybrid capsid of rAAV9 and rAAVrh74 that can specifically bind to the skeletal muscle-enriched receptor integrin alpha V beta (AVB6). One resulting variant, LICA1, had significantly enhanced infectivity in human myotubes and the skeletal muscle of a mouse model of Duchenne Muscular Dystrophy (DMD).^78^ Alternatively, chemical modification of the capsid can be achieved through genetic code expansion, a versatile platform to incorporate non-canonical amino acids (ncAAs) with desired properties into proteins at a specific site in a gene of interest.^79^ A recent study using this approach to engineer a novel class of rAAV, termed Nε-AAVs, by inserting single ncAAs via engineered orthogonal prokaryotic tRNA/tRNA synthases.^80^ These mutant Nε-AAVs were successfully conjugated with functional molecules. In vivo studies demonstrated that the functional molecule conjugated to Nε-AAVs resulted in highly specific uptake in the target cells in xenograft animal models. Another study also showed that the incorporation of a ncAA at D374 in the AAV5 capsid led to enhanced transduction specifically in mouse lungs.^81^ Despite these advances, rational design approaches are limited by our incomplete understanding of rAAV structure and biology.

##### Directed evolution

Directed evolution is a form of selective pressure to isolate capsid variants with prevailing properties, such as increased rAAV yield, enhanced transduction, immune response evasion, or specific cell/tissue tropism.^16^ One directed evolution strategy involves inducing random, unbiased mutations and subjecting capsids to selective pressure for viral fitness. This has been implemented by using error-prone PCR followed by a staggered extension process to generate a library of *Cap* mutants of VP1 – 3 to identify variants with altered affinities for heparin for immune escaping.^82^ In addition, a DNA shuffling-based approach has been utilized to generate diverse and extensive libraries of random chimeras by combining capsids from various parent AAV serotypes. This technique has led to the creation of new variants that demonstrate a wide range of cell tropism both in vitro and in vivo.^83,84^ Saturation mutagenesis of different antigenic footprints were also exploited to engineer an AAV8-derived capsid to evade NAbs and improve liver tropism.^85^

Surface panning of random peptides on an AAV capsid is another approach for directed evolution. For instance, a Cre recombination-based AAV targeted evolution (CREATE) system allows for attaching short-length peptides to rAAV9 capsid to engineer its properties.^86^ CREATE was used to generate AAV-PHP.B, an AAV9 variant that can cross the BBB to transduce the CNS in C57BL/6 mice.^86^ However, follow-up studies demonstrated that the properties of PHP.B capsid did not translate to other mouse strains or NHPs, suggesting a complex interplay between the engineered capsid and host factors which can differ between species.^87,88^

Subsequently, a novel rAAV evolution platform termed TRACER (tropism redirection of AAV by cell-type-specific expression of RNA) was developed. This platform is based on the recovery of viral RNAs expressed from a bulk capsid library carrying random peptide insertions in a cell-type-specific manner from animal tissue.^89^ A study in mice with peptide libraries displayed on rAAV9 capsids identified ten leading variants with up to 400-fold higher brain transduction over the parental rAAV9 following systemic administration. The same group recently reported that VCAP-102, a TRACER-engineered rAAV9 variant, displayed 20–90-fold increased transduction in diverse brain regions of NHPs compared to rAAV9.^90^ Iterative evolution of VCAP-102 led to capsid variants with 6–7-fold further improved BBB penetration. Similarly, TRACER-developed VCAP-100 derivatives showed up to 300-fold liver detargeting and six-fold higher brain transduction in NHPs over its parental AAV5.^91^

Directed evolution of rAAV capsids leveraging in vivo expression of transgene RNA (DELIVER) is another landmark method, which has led to the identification of highly functional muscle-tropic capsids in mice and NHPs.^92^ Cross-comparison of muscle-tropic variant capsid sequences in mice and NHPs identified a common RGD motif. Further analysis suggested that RGD-binding integrin heterodimers expressed in the target cells have a strong interaction with the viral variants containing the RGD motif. Researchers recently generated an RGD-embedded 7-mer peptide library inserted in variable region VIII of the capsid and identified variants with more than 20-fold enhancement in skeletal muscle transduction compared to the benchmark capsid.^93^

Directed evolution is a powerful tool to generate and identify variants with specific properties, but these properties are not always cross-species translatable in primates. For example, AAV-PHP.B showed enhanced CNS transduction in mice but not marmosets compared to AAV9, suggesting the necessity for cross-species evolution in capsid engineering.^94^ Nevertheless, a study sequentially evolving an rAAV capsid library across multiple species identified rAAV.cc47 as a potent cross-species variant with enhanced transduction capabilities over rAAV9 in the NHP brain and heart.^95^ 7m8 is an AAV2 variant with a 7-mer insertion to strongly transduce the outer retinal layers such as photoreceptor and retinal pigment epithelium (RPE) cells through intravitreal injection mainly in mice.^96^ A phase II clinical trial (NCT04418427) using ADVM-022 (AAV.7m8-aflibercept) for treating diabetic macular edema (DME) was suspended due to severe adverse effects in several patients 16–36 weeks after receiving high doses (6.0 × 10^11^ vector genomes (vgs)/eye), including refractory intraocular pressure reductions, underscoring the translational challenges of engineered capsid in the field.

##### In silico- or ML-based design

Computer-assisted rAAV engineering represents a cutting-edge approach that utilizes computational tools to enhance the design and optimization of rAAV. Studies have explored rAAV’s diverse properties from its evolutionary lineage and used reconstruction of the predicted ancestral genome to create novel capsid variants.^55,97^ Two similar studies computationally established the ancestral capsid library by rational design^55^ or adopting directed evolution.^97^ Both studies produced capsid variants with increased thermostability, an indicator of serotype identity, and promising biological properties desired for clinical application. Among these variants, Anc80L65,^55^ emerged as a promising vector for gene therapy for diseases associated with the inner ear^98,99^ or the eye.^100^

ML is extensively used in biomedical research, including medical image analysis, genetics, and drug discovery.^101,102^ A pioneering study applied ML to understand the rAAV2 capsid.^23^ They scrutinized the capsid’s fitness landscape, characterizing single-codon substitutions, and employing ML to design precise multi-mutated variants based on their impact on target tissues. Furthermore, deep learning has been used to diversify rAAV2 capsid variants, accurately predicting their viability.^103^ This approach reveals new possibilities for engineering the rAAV genome beyond the capsid, which has promising innovative applications in future rAAV development.

#### Engineering the cis-regulatory components of rAAV genome for transgene expression

The rAAV genome consists of ITRs and a transgene expression cassette consisting of the gene of interest and regulatory components. Strategies to engineer the rAAV genome include modifying ITRs or introns as well as inserting tissue-specific promoters for transcriptional regulation and/or using an inducible expression system, codon optimization, and CpG motif reduction of the transgene cDNA, and microRNA (miRNA)-mediated post-transcriptional regulated retargeting.^104–107^ Each of these components has its own functions, and their individual and collective actions determine transgene expression, cell-type specificity, safety, and durability of rAAV transduction.

##### Engineering rAAV ITRs

Traditional rAAV carries a single-stranded vector genome. Its transduction efficiency and rate depend on the second-strand synthesis of vector DNA before mRNA transcription occurs, which is the rate-limiting step. To increase transduction efficiency, strategies have been employed to mutate one of the ITRs, generating scAAV with a double-stranded vector genome.^53,54^ This allows scAAV to bypass the rate-limiting step of dsDNA synthesis, resulting in more rapid and efficient transduction of target cells.^20,108–110^ However, using scAAV has notable limitations: smaller packaging capacity (2.5 kb in scAAV vs 4.7 kb in ssAAV) and elevated risk of immune responses due to rapid accumulation of transgene products.^111,112^

The ITRs can be altered to selectively limit encapsidation to either the positive or negative strand of the vector genome.^113^ This is accomplished by selectively removing the D sequence from one of the two flanking ITRs. ITRs also harbor CpG motifs, which can signal to toll-like receptor (TLR)−9 and induce inflammatory responses. Complete removal of CpG from ITRs did not adversely affect vector genome copy number or transgene expression in treated animals. However, it resulted in a reduced rAAV titer, and whether it diminished inflammatory responses against rAAV remains unclear.^114^ Overall, ITRs are naturally prone to mutations and can be heterogeneous due to their palindromic nature, high GC content, and secondary structure. Optimizing the GC content in the ITRs could be a potential engineering strategy, ultimately improving rAAV properties.

##### Optimizing the promoters

Promoters in transgene expression cassettes are selected and optimized for specific needs such as tissue specificity, and expression level. The cytomegalovirus (*CMV*) or chicken beta-action (*CBA*) promoters are commonly used because they offer strong and ubiquitous expression, while tissue-specific promoters such as *CK8* or *MHCK7* provide targeted gene expression in muscle.^92^ The specificity of transgene expression within targeted tissues is crucial in order to achieve sufficient transduction efficiency with low dosing and minimize off-target effects. For example, a study employed a scAA9 vector carrying an endogenous human survival motor neuron 1 (*SMN1*) promoter to drive *SMN1* expression specifically in neurons.^115^ This approach demonstrated a remarkable safety profile, notably decreasing liver toxicity, and enhanced therapeutic efficacy in the SMNdelta7 mice with spinal muscular atrophy (SMA). The outcome surpassed the performance of Zolgensma, an FDA-approved treatment utilizing the *CMV/CBA* promoter, thus underscoring the advantages of tissue-specific promoters. Additional regulatory elements such as upstream enhancers can be engineered to improve expression activity and specificity. Numerous enhancers have been designed in clinical gene therapy vectors with the goal of increasing transgene expression, thereby reducing the viral load.^116^ Another study reported that a fragment of the mouse methyl-CpG-binding protein-2 promoter enabled robust long-term expression specifically in neurons.^117^ ML-based multi-omics data allows to design of tissue-specific promoters, resulting in an over 1000-fold dynamic increase in transgene activity between on- and off-target tissues.^118^

##### Transgene expression regulation

*Transcriptional regulation:* The expression of the gene or protein of interest can be up or down-regulated to optimize and fine-tune the desired effect. The tetracycline-inducible system is a widely used strategy to achieve a specific level of expression, although it is unlikely to be translated to humans due to potential immunogenicity issues.^119^ Alternatively, a system was developed to use the immune suppressive drug rapamycin to activate responsive transcription factors delivered by two separate rAAVs.^120^ This strategy was subsequently optimized so that only a single rAAV is now required. This has been used to achieve inducible dose-response expression to rapamycin treatment for long-term expression of the desired transgene.^121^ Importantly, there was only minimal transgene expression without the administration of rapamycin, indicating that this could potentially provide a specific and safe regulatory system.

*Post-transcriptional regulation:* Another strategy is the use of riboswitches, which are non-coding RNAs that can bind specific metabolites and control gene expression.^122^ Riboswitches occur naturally, and the principles of ligand-mediated gene regulation are being exploited by engineering aptazymes for mammalian systems with improved regulatory ranges. Using riboswitches over other regulatory systems has the advantages of being compatible with FDA-approved drugs, the small size of the RNA, and little or no immunogenicity. Importantly, riboswitches have very short sequences, which is particularly important when combined with the limited packaging capacity of rAAVs, making it a powerful system for regulating rAAV gene therapy.^123,124^ Non-coding RNAs are highly versatile tools in transgene regulation, which is further highlighted by incorporating RNA aptamers with cleavable poly A signal (PAS) into the 5′-untranslated region (UTR) of the transgene. In the absence of a small molecule, PAS cleavage leads to mRNA degradation to silence transgene expression. The addition of the small molecule leads to transgene expression through maintaining the integrity of the mRNA, which can be combined with alternative splicing to more precisely control gene expression.^125^

Some regulatory systems require either exogenous addition or co-expression of certain elements to work correctly. The use of drug-inducible splicing as a mechanism of gene expression regulation has been pursued to mitigate this problem. This approach entails the delivery of a gene containing a premature stop codon to prevent the translation of the protein. The addition of a small molecule splicing inducer facilitates the inclusion of a specifically designed exon into the mature transcript and the exclusion of the stop codon, thereby enabling the translation of the protein of interest.^126^ The strength of the promoter and dose of the inducer molecule allows for the expression level of the target gene to be precisely activated. However, current technologies rely on intrinsic protein turnover mechanisms to reduce levels of the target protein.

*Post-translational regulation:* One strategy to modulate the half-life of a protein of interest is the use of a protein degrading system (degron). This allows for a reversible and rapid response to protein removal and recovery. Several of these systems have been established in vitro, but attempts to translate this to in vivo systems have found high levels of toxicity. However, optimizations have generated newer, promising systems for in vivo applications, such as incorporating a SMAsh tag on the protein of interest. A small molecule drug that has already been approved for human use is then introduced to prevent the detachment of the SMAsh tag, which leads to protein degradation.^127^ Combining inducible expression systems, alternative splicing strategies, and protein degradation approaches has the potential to allow for exquisite control of a target protein’s expression level.

##### Optimizing the transgene (post-transcriptional level)

One common method in expression cassette engineering involves codon optimization by using computer algorithms to identify rare or suboptimal codons in the transgene to be replaced with preferred ones. Studies have demonstrated significantly higher expression levels in codon-optimized transgenes such as *FVIII* and the human cystic fibrosis transmembrane conductance regulator (*CFTR*) in mouse models.^128–131^ Moreover, a codon-optimized transgene of aspartoacylase (*ASPA*), the causative gene of Canavan disease, remarkedly restored ASPA protein expression and rescued the lethal disease phenotype in a mouse model.^132^ While codon optimization can be an effective strategy for improving the expression of the transgene delivered by rAAV, such effects can vary depending on the specific transgenes and the host organism. Codon optimization methods might introduce unexpected immunogenicity and toxicity as well as impair other traits such as tropism. Indeed, a study found that codon-optimization introduced a large quantity of CpG motifs into a rAAV8-based FIX Padua gene therapy for patients with hemophilia B, which could lead to innate immune responses.^133^ A novel recurrent neural network-based tool was developed to optimize codon usage for specific cell types using data from mouse myocytes, neurons, and hepatocytes. This approach improved protein expression and reduced CpG dinucleotides, holding promise for enhancing tissue-specific gene therapy efficiency.^134^

##### Modifying other cis-regulatory elements

Other *cis*-regulatory elements can be modified in the expression cassette to adjust gene expression and specificity. For example, the woodchuck hepatitis virus post-transcriptional regulatory element (*WPRE*) is a ~600 bp RNA element that is commonly added downstream of the transgene. The *WPRE* can enhance transgene expression both in vitro and in vivo by increasing the amount of mRNA transcribed independent of the transgene.^135,136^ Studies have cautiously reported potential oncogenic activity of the *WPRE* incorporated in lentivirus vectors delivered to mice.^137,138^ However, such risk can be reduced by removing the oncogenic sequence from the original *WPRE*.^137^

The inclusion of an intron in a transgene expression cassette can also improve gene expression in animals.^139^ This was supported by another study which showed that an intron increased transgene expression by 40–100-fold in vivo.^140^ However, the effects of introns on gene expression can be complex and depend on a variety of factors, such as the size and location of introns and the specific target cells. The selection of a proper polyA signal is important for optimizing transgene expression and stability (e.g., beta-globin, SV40, or bovine growth hormone (BGH)). A comparison study found that a modified shorter version of SV40 late polyA (135 bp) showed comparable transgene expression to a BGH polyA (223 bp) in a mouse brain.^141^ In addition, the inclusion of a Kozak sequence can further enhance the transgene expression.^132^

Ensuring confined transgene expression to target tissues is vital to avoid toxic overexpression and immunotoxicity. rAAV design incorporates cell-specific promoters as a primary strategy for targeted expression, and fine-tuning strategies are then developed such as adding miRNA binding sites in the 3′-UTR to inhibit expression in cells expressing the complementary miRNA. For example, adding miR-122 binding sites in rAAV9 was reported to enable CNS expression while retargeting liver, heart, and skeletal muscle.^104^ When rAAV carries a foreign transgene or one encoding a replacement gene in patients who have null mutations in the endogenous copy, the transgene product may be regarded as a non-self-antigen by antigen-presenting cells (APCs), which then process the transgene for major histocompatibility complex (MHC) presentation and immune clearance. One strategy to mitigate transgene immunity is to incorporate APC-specific miRNA binding sites, such as miR-142 and miR-652, into the expression cassette of rAAV to detarget transgene expression from APCs.^142,143^ The results showed a decrease in transgene-specific immune responses and sustained transgene expression in targeted cells in mice. Such a strategy has been translated in NHPs, in which broadly NAbs against human immunodeficiency virus were durably expressed after delivery by rAAV, thus highlighting this strategy’s potential for clinical translation.^144^

#### Expanding rAAV packaging capacity

Another significant challenge in rAAV application is the delivery of transgenes that surpass its 4.7 kb packaging capacity. Examples include therapeutic transgenes like centrosomal protein (*CEP290*) for Leber’s congenital amaurosis type 10 (~7.5 kb),^145^ or CRISPR-based tools like cytosine base editor or prime editor 2 (>5 kb).^146,147^ Various strategies have emerged to overcome this hurdle by dividing a large transgene into two parts, with each encapsulated in an rAAV capsid. Co-introduction of these segmented transgenes into the same cell results in the reconstitution of the full-length transgene and protein. This reconstitution can occur at different stages within the genetic information flow.^19^ At the DNA level, inter-vector DNA recombination is facilitated by rAAV ITRs, a partial transgene sequence, or an optimized recombinogenic sequence present in both rAAV genomes.^148–150^ The overlapping sequence can be excised post-transcription through specifically designed splicing signals, resulting in a mature full-length mRNA that is subsequently translated into the desired protein. At the RNA level, trans-splicing between two transcripts from individual vectors, mediated by the splicing donor and acceptor present in each transcript, generates a full-length transcript and protein.^151–153^ At the protein level, protein reconstitution has been achieved through split inteins, the natural polypeptides capable of excising itself and ligating the nearby protein segments.^154–160^

## rAAV manufacturing

### **Methodologies**

**Fig. 4:**
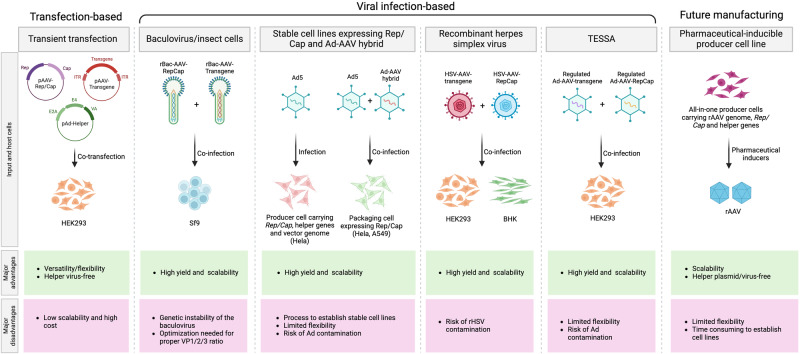
Current approaches to manufacture rAAV. Currently, two main platforms are used for rAAV manufacturing: transfection- and viral infection-based approaches. Plasmid transient transfection of HEK293 cells remains the most widely used method, while stable cell lines, baculovirus (BV) systems, and the HSV type I system offer scalable alternatives for large-scale production. The transfection-free helper virus system TESSA has been developed to produce high-yield rAAVs. Pharmaceutically inducible all-in-one producer cell lines may represent the next generation’s optimal manufacturing platform for rAAV-based drugs. BHK baby hamster kidney. Figure created with Biorender.com

The clinical applications of rAAV highlight the urgent demand for production and purification systems capable of producing substantial quantities and high-quality rAAVs to meet safety, efficacy, stability, and cost requirements (1.0 × 10^15^–1.0 × 10^16^ vgs per treatment).^161^ Because rAAV is unable to self-amplify by reinfection, its production requires the simultaneous expression of helper viral genes and rAAV-specific *Rep* and *Cap* genes for replication and packaging of the ITR-flanked vector genomes. Methods for rAAV production have been developed primarily with two strategies: transient transfection and viral infection. For transfection-based rAAV production, plasmid co-transfection into HEK293 cells is the most widely used production system, which was pioneered by Xiao et al. ^29^ rAAV production through viral infection involves the use of three different viruses, including AAV’s natural helper viruses, Ad and HSV, and baculovirus are used. rAAV production also involves either mammalian (e.g., HeLa, A549, and HEK293) or insect cells (e.g., *Spodoptera frugiperda*, Sf9) (Fig. 4).^5^ A newly developed tetracycline-enabled self-silencing Ad (TESSA) represents another infection-based system for large-scale, high-yield rAAV production in HEK293 cells.^162^ Each of these methods has its unique advantages and drawbacks in terms of flexibility, quality, and scalability, which will be discussed in more detail below. To purify rAAV produced by various methods, gradient sedimentation using either cesium chloride or iodixanol gradient ultracentrifugation is a commonly employed generic procedure for small-scale rAAV preparations.^163–165^ For large-scale vector production, and particularly for clinic-grade vectors that demand higher quality and quantity, affinity- and/or ion-exchange-based column chromatography is required.^166^ Finally, the development of stable packaging and producer cell lines is being actively pursued as the preferred next-generation rAAV production platform due to their straightforward scalability for large-scale production at a lower cost.

#### Transient transfection in HEK293 cells

Traditional rAAV production involves transfecting three plasmids carrying vector genome, AAV *Rep*/*Cap* genes, and Ad helper genes (*VA RNA*, *E2A*, and *E4OEF6*), typically at equimolar ratios, into HEK293 cells. Another essential Ad helper gene, *E1a/b*, is inherently expressed in HEK293 cells.^167,168^ This triple transfection protocol was subsequently optimized to a two-plasmid co-transfection system, involving one plasmid for the rAAV genome and another for *Rep*/*Cap* and Ad helper genes.^169,170^ Current methods can achieve approximately 80% cell transfection rate with peak virus yield at 48–72 h post-transfection.^171^

There are numerous challenges in viral vector manufacturing that lead to inconsistencies in quality, titers, and purity between batches,^172^ and the process is very labor-intensive.^173^ Recent research has focused on modifying the standard manufacturing process at the plasmid level to enhance both yield and purity with reduced cost. One notable recent study showed that low-cis triple transfection (1% or 10% cis plasmid) significantly reduced the transgene plasmid usage compared to the standard triple transfection (100% cis plasmid) and increased the in vivo transduction efficiency.^174^ Importantly, this method enables the packaging of yield-inhibiting transgene and reduces plasmid backbone contamination in both low-cis rAAV preparations and tissues of mice receiving this rAAV. Others developed a new all-in-one rAAV production system termed AAVone that combines *Rep/Cap* and Ad helper genes with the rAAV genome into a single plasmid. AAVone yields higher rAAV production compared to the triple transfection method, with lower batch-to-batch variations and reduced levels of replication-competent AAV (rcAAV). It also requires less plasmid DNA and eliminates the need for ratio optimization steps.^175^

The transient transfection approach presents flexibility by enabling the easy and rapid generation of rAAV with diverse transgenes and capsids. It can be scalable to an extent when using suspension cells, allowing for production to be increased simply by increasing the culture volume. In addition, the transient transfection method is flexible and versatile, and does not require time-consuming processes of establishing and maintaining stable producer cell lines for specific vector products. This facilitates a quicker turnaround time from vector design to production. Notably, this approach suffers from limited scalability and high cost.

#### Baculovirus production system

The first rAAV gene therapy product to reach the market (uniQure’s Glybera,^176^ alipogene tiparvovec; now withdrawn from the market) actually utilized the Baculovirus Expression Vector Systems (BEVS), in which baculoviruses encoding *Rep* and *Cap* genes and carrying rAAV genome infect insect cells, such as Sf9, to produce rAAV.^177^ This system was originally optimized for large-scale protein production, but significant advancements have been made to accommodate for rAAV production. The first-generation BEVS involved splitting the *Rep* gene into two cassettes controlled by different promoters and modifying the *Cap* gene to include a non-canonical start codon (ACG instead of AUG). These genetically altered AAV viral sequences were incorporated into three separate baculovirus constructs, necessitating simultaneous superinfection of all three in a single cell.^177^ However, this method was initially successful for rAAV1 production but much less productive for other rAAV serotypes. Second-generation BEVS was more adaptable for additional rAAV serotypes and reduced the number of baculoviruses to two, one carrying *Rep*/*Cap* genes (Bac-CapRep) and another carrying the rAAV genome (Bac-Transgene).^178^ Subsequently, an attenuated Kozak sequence and leaky ribosome scanning were introduced to BEVS to ensure proper AAV gene expression, resulting in rAAV with significantly improved biological potency.^179^

Recently, BEVS has been further improved so that only one recombinant baculovirus is required for rAAV production. An engineered Sf9 cell line was created by integrating *Rep*/*Cap* genes, which then only needs one infection by a baculovirus carrying an ITR-flanked transgene, resulting in higher rAAV yields compared to the conventional three-Bac system.^180^ Another advancement introduced a highly regulated *Rep* gene control system to stabilize and allow appropriate Rep expression, leading to the production of potent and high-yield rAAV particles.^181^

One of the drawbacks of BEVS is the genetic instability of baculovirus. Baculoviruses naturally remove parts of their genomes during replication, which generates mutant viruses, some of which can become defective interfering particles.^182,183^ During the scaling-up process in cell culture, these mutants may outcompete baculoviruses with intact genomes, potentially reducing rAAV production. However, the genetic instability of baculoviruses can be potentially improved by either deleting specific genes or inserting transgenes into stable loci within the baculovirus genome.^184,185^ Additionally, BEVS is inherently not capable of assembling capsid proteins into AAV virions with the VP1, VP2, and VP3 at a ratio of 1:1:10, causing a reduction of transduction potency for the resulting rAAVs. A study demonstrated that the incorporation of artificial introns within the rAAV sequence, such as those using the baculoviral polyhedrin promoter, yielded infectious rAAV with high titers across various serotypes, reaching up to 1.0 × 10^14^ vgs/L.^186^ Furthermore, a study systematically compared rAAV produced by BEVS (Sf9-rAAV) to that produced by transient transfection (HEK-rAAV) using large-scale suspension cultures and found that Sf9-rAAV may be favored over HEK293-rAAV for its superior yields, full/empty ratio, scalability, and cost-effectiveness.^187^ However, further studies into the genetics and biology of baculovirus are necessary in order to harness the advantages of the BEVS platform (Fig. 4), such as its high scalability.

#### Mammalian stable cell lines and Ad-AAV hybrid for rAAV production

Stable cell lines for vector production offer notable advantages, including easy product characterization, scalability, and the capacity to generate higher yields with improved reproducibility and reduced cost.^188,189^ There are two types of stable cell lines for Ad infection-based rAAV production: packaging and producer cell lines. Packaging cell lines have stably integrated AAV *Rep*/*Cap* genes. A major challenge for rAAV production using packaging cell lines is that the cells need to be first infected with AAV’s natural helper virus, wild-type Ad, to initiate endogenous Rep/Cap expression, followed by secondary infection with Ad-AAV hybrid virus from which rAAV genomes will be rescued, replicated, and packaged.^190,191^ In contrast, producer cell lines have *Rep/Cap* and Ad helper genes, as well as rAAV genome, stably integrated into the host genomes. In this case, infection by wild-type Ad will activate *Rep/Cap* gene expression, facilitating the rescue, replication, and packaging of rAAV genomes.^192^ Stable cell lines derived from A549 and HEK293 cells have been adapted for suspension cultures for scalable rAAV production.^193–195^

Several HeLa-based cell lines, such as C12, H44, and B50, yielded high titers of infectious and rcAAV-free rAAVs through endogenous expression of Rep/Cap. An A549-derived stable cell line expressing Rep/Cap and a K209 cell line has also been reported to produce high yields of infectious rAAV per cell.^193,196^

There are several drawbacks to stable cell lines and Ad infection-based rAAV production systems. First, creating stable cell lines is often a complex, time-consuming, serotype-, and vector genome-specific process. Second, ensuring the characterization and stability of these cell lines can be challenging, with potential risks related to the impact of passage history on growth kinetics. Additionally, to ensure the safety of the final gene therapy product, robust downstream purification procedures are essential to eliminate any pathogenic Ad contaminants and oncogenic HeLa DNA.^195^

#### Recombinant herpes simplex virus (rHSV) system for rAAV production

rHSV is another natural helper virus capable of providing helper functions for rAAV production.^197,198^ In order to produce rAAVs using an rHSV system, two distinct rHSVs carrying the AAV *Rep*/*Cap* genes and rAAV genome are created for sequential infections of HEK293 or baby hamster kidney cells, followed by downstream processing and purification. The first clinical trial using rAAV1 to treat α1-antitrypsin deficiency was produced using rHSV.^199^

The notable drawbacks of using the rHSV-based system for rAAV manufacturing include the generation of a sufficient quantity of rHSV particles and the specialized purification steps necessary to effectively eliminate neurotrophic and neurotoxic rHSVs and other associated contaminants from the final rAAV preparations.

#### Tetracycline-enabled self-silencing adenovirus (TESSA) system

Typical methods for rAAV production involve either helper-free plasmid transfection or the use of a helper virus. However, the helper-free system poses challenges when scaling up and the helper virus approach requires more robust downstream processing and purification to eliminate contaminating helper viruses and highly immunogenic VPs.^195,200^ A recent innovation introduced a helper Ad designed to inhibit its major late promoter (*MLP*), thereby restricting its replication and effectively overcoming these limitations.^162^ This was accomplished by introducing an inducible tetracycline repressor binding site into the *MLP*. This modified Ad functions normally when doxycycline is present but is restricted to genome amplification and early gene expression (known as the helper functions) without it. Through the utilization of this self-regulating Ad, researchers successfully facilitated the delivery of essential adenoviral helper functions, *Rep*/*Cap* genes, and the rAAV genome, resulting in a substantial enhancement in rAAV production with a nearly complete absence of contaminating Ad. Previously, numerous attempts to generate a genetically stable recombinant Ad expressing the AAV *Rep* gene in HEK293 cells have failed due to the cytotoxic and recombinogenic nature of Rep protein even at a very low level of leaky expression.^201^ However, the TESSA system tightly regulates the expression of the *Rep* gene, activating its expression only during rAAV genome rescue, replication, and packaging process, which is a major breakthrough. This system improved the production of various rAAV serotypes in both HEK293 and U87 cells, including rAAV2, rAAV6, rAAV8, and rAAV9.^162^ Moreover, this system can be scaled up to produce rAAV2 and rAAV6 with a significant improvement on vector yield over the transient transfection approach.^202^

### Next-generation manufacturing platform technology

Selecting which production system to use involves balancing flexibility, scalability, and quality, all of which play a vital role in the development of a safe and effective rAAV product to meet the demand, safety, and efficacy requirements for clinical applications.

To further improve the transient transfection approach, it is crucial to optimize multiple factors during both the production and purification processes, aiming for increased yield and reduced empty rAAVs. Minimizing empty vectors enhances vector potency, which allows for a lower injection dose to be used, thereby reducing immunotoxicity. Ongoing efforts are underway to optimize the upstream processes of rAAV production to improve rAAV yield. For example, adjusting the plasmid ratio by reducing the cis plasmid to 1% or 10% can achieve a yield of rAAV that is comparable to conventional triple transfection. This approach enables the packaging of previously unpackageable transgenes while also reducing backbone plasmid contamination and is proving to be a cost-effective strategy.^174^

A mechanistic modeling study using triple transfection has identified several key bottlenecks in rAAV production, including inefficient plasmid delivery to the nucleus and poorly coordinated timing of capsid synthesis and viral genome replication leading to the empty capsid.^203^ Strategies to address these issues include ensuring that viral DNA replication occurs before or in parallel with capsid production, such as early expression of Rep protein or late expression of Cap protein.

Efforts have also been made to identify small-molecule chemical additives that can boost rAAV production.^204–206^ A library of over 130 small molecules has been assembled to transiently antagonize a broad range of cellular innate antiviral pathways, effectively increasing viral production at scale.^205^ Imidazole-based small molecules have been shown to improve large-scale rAAV production through multiple signaling pathways.^206^

Modifying host factors can also affect production. Through a genome-wide screening strategy, studies identified several gain- or loss-of-function targets that can significantly affect rAAV production.^207,208^ Indeed, a systemic analysis of the cellular transcriptional response to transient plasmid transfection revealed that host cells actively sense rAAV production as an infectious insult and upregulate inflammatory and antiviral responses.^209^

To reduce empty AAV particles, a study created a hybrid *Rep* gene from different AAV serotypes with the 3′ end of the AAV2 *Rep* gene, resulting in a 2–4-fold increase in full capsids for non-AAV2 serotypes, suggesting that enhancing Rep function may improve rAAV production.^210^

Efforts in optimizing downstream purification processes have also led to improvements in removing empty capsids. The industry has transitioned from ultracentrifugation to column-based chromatography for effective purification of rAAV particles from impurities in large-scale production.^211^ Anion-exchange chromatography has become a major focus in the field, with modifications to various process parameters, including pH, various elution salts, excipients, surfactants, stabilizers, and osmolytes to enhance purification efficiency.^212^

Future manufacturing approaches should prioritize maximum flexibility, scalability, and quality while eliminating the need for plasmid transfection, virus infection, and chemical inducibility. Numerous efforts have been directed toward creating a stable and inducible producer cell line with these essential characteristics. One notable study exemplifies this approach by integrating all components necessary for rAAV production into HEK293 cells, including the rAAV genome, *Rep/Cap* genes, and helper coding sequences sophistically controlled by multiple inducible promoters. The resulting synthetic cell line generates infectious rAAVs upon induction.^213^ This all-in-one producer cell line approach allows for independent control over replication and packaging activities, ensuring high-quality rAAV production. Subsequent work has further optimized this producer cell line to enhance productivity by reducing the overexpression of the gene of interest, optimizing induction profiles, and mitigating proteasomal degradation of capsid protein by proteosome inhibitors.^214^ For large-scale vector production, another two-step platform for generating a stable and inducible producer cell line has been developed. Initially, an alpha cell line is created based on a proprietary CAP human cell line or HEK293 cells in suspension with stably integrated *Rep* and helper genes. This is followed by the integration of the *Cap* gene and the gene of interest, resulting in a polyclonal producer pool, followed by a single-cell cloning process, clone screening, and selection of the best-performing clone.^215^ rAAV production in this system is induced by doxycycline. In a proof-of-concept cell line with perfusion bioprocessing for rAAV8-GFP production, the rAAV per cell yield was enhanced 8-fold, with a significantly increased yield of full particles (30–40%) compared to the conventional batch process.

Enhancing vector genome integrity is another strategy to increase vector potency. A study using a long-read sequencing-based platform and bioinformatic pipeline identified different vector genomic populations from scAAV preparations.^216^ It concluded that robust DNA secondary structures inherent in vector genome designs can cause a significant truncation, impacting both production and vector efficacy. Another study reported the presence of truncated genomes and a unique genomic species that originated from a nickable ITR incorporating a small portion of payload and a chimeric sequence that joined to the plasmid backbone.^217^ This heterogeneity was also observed in a vector genome design carrying dual single-guide RNA expression cassettes in tail-to-tail configurations.^218^ Interestingly, the same group found notable differences in genome heterogeneity between human and insect cell-produced rAAV.^219^ These findings highlight the urgent need to scrutinize vector genome design and generation of heterogeneity for the efficient production of highly potent rAAV preparations, particularly for clinical vectors.

## Gene therapy efficacy and clinical applications

### **Overview of current clinical trials of rAAV-based gene therapy**

**Fig. 5:**
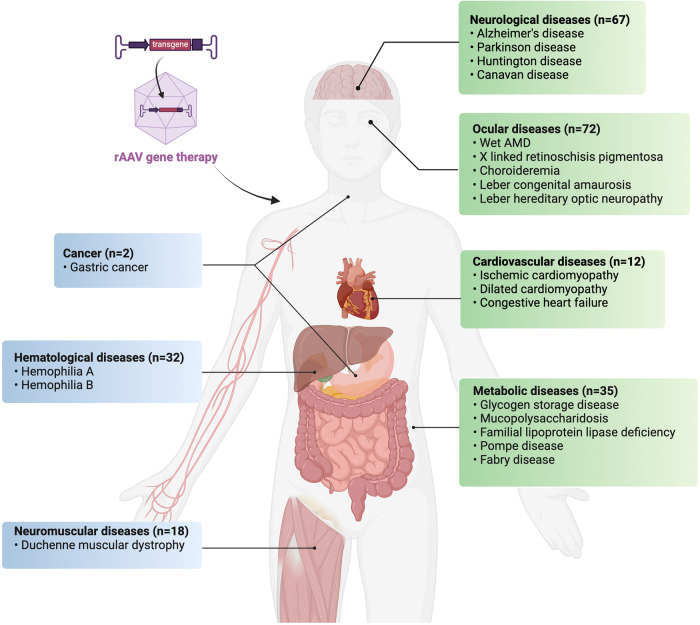
Current clinical applications of rAAV in major human diseases. Clinical applications of rAAV across a spectrum of significant human diseases, including ocular, neurological, metabolic, hematological, neuromuscular, cardiovascular diseases, and oncology. Refer to Supplementary Table 1 for a comprehensive list of 238 clinical trials employing rAAV-based gene therapy for the aforementioned diseases. Figure created with Biorender.com

There have been remarkable developments in the clinical use of rAAV over the last few decades, underscoring its tremendous potential in gene therapy for a wide range of genetic and acquired diseases. Indeed, in a relatively short timeframe since the FDA approved Luxturna,^220^ there have been five additional rAAV gene therapy products introduced to the market (Fig. 1). Although rAAV-based therapies have shown impressive results in some clinical trials, numerous aspects require further investigation, including vector immunogenicity, dose optimization, and long-term safety. While meeting high-yield manufacturing challenges may not be an immediate issue for most monogenic diseases, producing rAAV at a large scale could become a bottleneck as more rAAV gene therapies are being developed for treating chronic prevalent human diseases. Furthermore, the regulatory framework for gene therapies is undergoing rapid changes. In this section, we will explore the latest developments in clinical applications of rAAV in major human diseases (Fig. 5), including ocular, neurological, metabolic, hematological, neuromuscular, cardiovascular, and oncogenic diseases. A table summarizing 238 clinical trials of rAAV-based gene therapies is presented in Supplementary Table 1.

#### Ocular diseases

Ocular diseases are at the forefront of rAAV gene therapy for several reasons. The eye’s immune-privileged status reduces immune responses to rAAV. Its small volume necessitates low rAAV doses. Many ocular diseases are monogenic, making them suitable for gene therapy. Additionally, the relatively easy accessibility of the eye allows for various rAAV administration routes. Here, we highlight several rAAV-based ocular gene therapies, focusing on the treatment of monogenetic and acquired ocular diseases.

##### Leber congenital amaurosis type 2 (LCA2)

Voretigene neparvovec-rzyl (Luxturna) is a gene therapy to treat LCA2, a rare form of inherited retinal disease (IRD) caused by mutations in *RPE65*. This therapy delivers a functional copy of the *RPE65* gene, correcting the deficiency in RPE cells, which are crucial for visual acuity. In a landmark phase III clinical trial, 29 LCA patients with confirmed biallelic *RPE65* mutations were randomized to receive either Luxturna or no treatments as controls, followed by multi-luminance mobility testing, a measurement of visual function at specific light levels by requiring participants to navigate an obstacle course.^221^ The results indicate that Luxturna significantly improved functional vision (average of 1.8 light levels) compared to controls (average of 0.2 light levels) within one year. Moreover, full-field sensitivity threshold testing showed greater than 100-fold improvement in the intervention group by day 30, which was maintained over a year. Such beneficial improvements were sustained over 3–4 years in the subsequent follow-up studies.^222,223^ Due to the favorable results observed in this phase III trial, Luxturna was approved by the FDA in 2017 for the treatment of biallelic *RPE65* deficient-associated retinal disease as the first gene therapy for genetic diseases.^220^

##### Retinitis pigmentosa (RP)

Given the heterogeneity of causative mutations in RP, various gene therapy strategies have been applied to this common form of IRD. Loss of *MERTK*, a crucial component in a signaling pathway that controls phagocytosis in the RPE, leads to photoreceptor degeneration and ultimately RP.^224^ This disease phenotype is autosomal recessive and accounts for ~3% of RP cases. A phase I clinical trial assessed subretinal injection of rAAV2-VMD2-*hMERTK* in six patients with *MERTK*-associated RP.^224^ While three patients showed vision improvement, sustained visual gains were only observed in one patient over 2 years, indicating that long-term investigation is needed.

Retinitis pigmentosa GTPase regulator (*RPGR*) gene mutation is a common cause of X-linked retinitis pigmentosa (XLRP) and primarily affects males. This is the most severe form of RP and is characterized by progressive vision loss due to dysfunction of photoreceptors and RPE cells.^225^ Three clinical trials have been conducted to assess the safety and efficacy of rAAV-based gene therapy to treat patients with *RPGR* mutations.^226–228^ Early results from one of the trials are encouraging, with some patients showing improvement in visual function and stability of retinal structure after receiving the treatment.^226^ A post hoc analysis of the gene therapy clinical trial (XIRIUS) and natural history study (XOLARIS) for XLRP revealed that the four participants who received the highest doses of cotoretigene toliparvovec (BIIB112/rAAV8-*RPGR*) had early improvements in retinal sensitivity and low-luminance visual acuity at month 12.^229^ In a phase II/III study, however, this gene therapy did not meet the primary endpoint of statistically significant improvement in light sensitivity in treated eyes.

Approximately 15% of RP cases are caused by autosomal dominant gain-of-function rhodopsin (*RHO*) mutations.^230^ Gene silencing or knock-out strategies can be used for this type of mutation. A preclinical study was conducted in NHPs using EDIT-103,^231^ an investigative gene therapy product containing two rAAV5 vectors to remove endogenous *RHO* through CRISPR/Cas9 and insert a codon-optimized *RHO* expression cassette that is resistant to targeted Cas9 cleavage to restore functional RHO expression. Early results have shown an encouraging improvement in RHO expression and visual function. However, its efficacy in humans is yet to be assessed.

In addition to gene replacement or editing approaches, other innovative strategies such as optogenetics have been investigated to treat RP independently of the genetic mutation. Optogenetics uses light to manipulate genetically modified cells with light-sensitive proteins. The initial assessment from a phase IIb clinical trial concluded that intravitreal injection of MCO-010, a multi-characteristic opsin encoded in rAAV2 designed to specifically target ON retinal bipolar cells, in patients with RP resulted in vision improvement with no severe ocular or systemic adverse events.^232^

##### Age-related macular degeneration (AMD)

AMD is the most common cause of blindness in developed countries. It is a progressive retinal condition featuring geographic atrophy and neovascularization at late stages. The mainstay of the treatment is to target vascular endothelial growth factor (VEGF), a major angiogenic factor involved in the pathogenesis of neovascularization. While anti-VEGF treatments have been proven to be efficient in alleviating disease progress and improving visual function in some patients, they require monthly injections and thus cause a high treatment burden while proving unsuccessful for a large proportion of patients. Gene therapy enables long-lasting, endogenous production of a therapeutic protein, solving many limitations of anti-VEGF agents, such as its short half-lives. As a result, there are an increasing number of clinical trials using rAAV to deliver genes for the treatment of both geographic atrophy and neovascular AMD.

GT005 (Gyroscope) is a rAAV2-based, one-time gene therapy product for geographic atrophy secondary to AMD that is delivered through subretinal injection. GT005 aims to mitigate retinal degeneration caused by an overreactive complement system in the aging retina by increasing the expression of the complement factor I (CFI) protein, a gene addition strategy to reduce inflammation and alleviate the degeneration of retinal cells. EXPLORE and HORIZON are phase II, multicenter, randomized, and controlled (treated vs. untreated patients) trials evaluating the safety and efficacy of GT005 in two different groups of patients with geographic atrophy (EXPLORE: patients with geographic atrophy secondary to AMD and with low CFI expression due to CFI variants; HORIZON: patients only with geographic atrophy secondary to AMD). Interim data indicate that the treatment achieves a sustained increase in vitreous CFI levels and a decrease in downstream proteins of CFI. The effect of the treatment is localized to the eye and no systemic increase in CFI levels has been observed. Nevertheless, the sponsor recently discontinued further development of GT005 because the futility criteria had been met.

sFlt-1 is a chimeric VEGF inhibitory protein consisting of domain 2 of Flt-1 (VEGF receptor-1) coupled to the human immunoglobulin G1 heavy chain Fc fragment.^233^ Gene therapy for neovascular AMD by using rAAV2 encoding *sFlt-1* has been investigated through both intravitreal and subretinal administration.^234–237^ Intravitreal injection of rAAV2-*sFlt-1* in a phase I clinical trial was reported to be safe and well-tolerated, while the expression level of sFlt-1 and the treatment efficacy was variable.^238^ Similarly, subretinal injection of the same vector was well-tolerated among the elderly.^239^ However, significant visual improvements were not observed, although this outcome was not the primary endpoint for the study.

RGX-314 (Regenxbio) is being developed as a subretinal treatment for neovascular AMD, consisting of rAAV8 encoding anti-VEGF monoclonal antibody fragments similar to ranibizumab. Initial assessment from a phase I clinical trial (ASCENT) showed that the treatment was safe and well-tolerated. The treatment outcome was dose-dependent, and several patients remained free of supplemental anti-VEGF injections for up to 18 months.

ADVM-022 (Adverum Biotechnologies) is a gene therapy product utilizing an engineered 7m8 vector derived from rAAV2 encoding aflibercept for the treatment of neovascular AMD. In a phase I clinical trial (OPTIC), a single intravitreal injection of ADVM-022 reduced the need for supplemental anti-VEGF injection in over 80% of patients for up to 92 weeks during the follow-up period.^240^

#### Neurological diseases

Current FDA-approved rAAV gene therapies for neurological disorders use two fundamentally different routes of delivery for localized versus widespread transgene delivery: stereotactic, anatomically confined rAAV delivery or widespread CNS transduction via intravenous (i.v.) delivery.

##### Aromatic L-amino acid decarboxylase (AADC) deficiency (AADCD)

AADCD is an autosomal recessive disorder caused by mutations in the dopa decarboxylase gene, leading to AADC enzyme deficiency.^241–243^ AADC catalyzes the final step in serotonin and dopamine synthesis, with dopamine as a precursor for norepinephrine and epinephrine. This leads to a combined serotonin, dopamine, norepinephrine, and epinephrine deficiency.^242^

Patients present during the first months of life with muscular hypotonia, dystonia, oculogyric crisis, developmental delay, and autonomic dysfunction.^241^ A substantial delay between symptom onset (2.7 months) and diagnosis (3.5 years) has been reported.^242^ In its most severe form, patients miss all developmental milestones, lack head control, never learn to stand or sit, and die before the age of 10 years.^241,244^

Parkinson’s disease (PD) and AADCD have different etiologies (neuronal loss vs. enzyme deficiency) but result in striatal dopamine deficiency as a shared therapeutic target. This led to the rationale that one therapeutic vector could benefit both diseases. The first attempt to deliver intraluminal (the putamen is part of the striatum) rAAV expressing AADC (rAAV.*AADC*) was tested in 2008 in PD patients.^245–247^ Overall, the therapy was well-tolerated, with few procedure-related adverse effects, providing critical data on the safety and feasibility of stereotactic intracranial rAAV.*AADC* delivery was also associated with clinical improvement.^246^ Encouraged by the PD trials, this approach was extended to children with AADCD in 2012 in Taiwan.^248^ Four children, with the oldest being six years of age at the time of treatment, received bilateral intraputaminal infusion of rAAV2.*AADC* at a total dose of 1.6 × 10^11^ vgs. All patients showed improved motor scores and subjectively reported improved emotional stability. Interestingly, patients experienced transient post-infusion dyskinesia.^248^

A follow-up phase I/II trial in 10 patients used a slightly higher dose (1.81 × 10^11^ vgs), with the oldest patient being 8 years of age at the time of treatment, and no immunosuppression was used. All patients met the primary endpoints of clinical motor performance and cerebrospinal fluid (CSF) biochemistry improvement. Other measures, such as positron emission tomography (PET) imaging, showed significantly improved AADC activity. Again, post-infusion-related transient dyskinesia was noted.^249^

In 2019, a group in Japan treated six patients in a phase I/II trial with the same approach and a similar vector and dose (2 × 10^11^ vgs) as the Taiwanese group.^250^ The patient population, however, was more diverse regarding mutations and age at treatment, with the oldest patient being 19 years old. Similar to the 2017 Taiwanese study, patients experienced transient post-infusion uncoordinated movements of the mouth and extremities. Encouragingly, all patients improved in motor performance and oral food intake.

The anatomical target was selected based on PD pathology defined by dopaminergic neuron loss. However, in AADCD, dopamine-deficient dopaminergic neurons seem to persist. Targeting these dopaminergic neurons in the substantia nigra and ventral tegmental area to emulate physiologic dopamine synthesis was recently evaluated in a safety and efficacy trial.^251^ Seven patients between 4 to 9 years of age were assigned to two dosing cohorts (8.3 × 10^11^ and 2.6 × 10^12^ vgs total dose). All patients experienced improved motor function and mood. In 6/7 patients, oculogyric crises ceased completely. This clinical improvement was consistent with PET imaging results suggesting AADC enzyme activity. As in most trials, transient dyskinesia was observed post-infusion. Unfortunately, a direct comparison of both approaches is difficult at this stage and requires further studies.

Recently, long-term follow-up results from the Taiwanese trials reported significant improvement in motor function in 26 patients, with three patients able to walk.^252^ Interestingly, treatment age negatively correlated with motor scores, while dose did not.^252^ The most common treatment-emergent adverse effects (TEAE) were pyrexia and dyskinesia. Dyskinesia resolved in all patients but one. Notably, a positive correlation between dyskinesia severity/duration with treatment age was suggested.^252^ This correlation could be a critical observation to understand age-risk-benefit and might find equivalents in other CNS diseases. The therapy (Eladocagene Exuparvovec) was eventually licensed and has recently been approved by the European Medicines Agency for patients over 18 months of age (Upstaza).^253^

##### Spinal muscular atrophy (SMA)

SMA is a model disease to targets widespread pathology in the brain and spinal cord by i.v. rAAV gene therapy. Over 3000 patients have been treated to date, providing critical insights into efficacy and safety. SMA is classified into variants I–IV.^254^ Variant I is the most severe form, with symptom onset in the first 6 months of life and death before the age of 2 years.^255,256^ It is characterized by weakness, motor development regression, and progresses to respiratory failure and death. SMA I is autosomal recessive and caused by loss-of-function mutations in the *SMN1* gene.^257^ *SMN2*, a gene homolog to *SMN1*, produces a short version of *SMN1*. The number of *SMN2* copies negatively correlates with the severity of the disease. The incidence varies based on country and ethnicity but is estimated as low as 1 in 7829.^258^

The first clinical trial results for SMA I were published in 2017.^259^ 15 patients received either 6.7 × 10^13^ vgs/kg or 2 × 10^14^ vgs/kg of i.v. rAAV9 expressing *SMN1* (rAAV.*SMN1*) under the control of the CB promoter with a CMV enhancer. The mean age was 6.3 months in the low dose and 3.4 months in the high dose cohort. The main findings were that patients had a reduced need for respiratory ventilator support and improved motor scores. 11/12 patients were able to sit unassisted for several seconds and gained the ability to speak. While the therapy was well-tolerated, elevated liver enzymes were common, but decreased with corticosteroid treatment.^259^

In two phase III follow-up studies in the US (STR1VE) and the EU (STR1VE-EU), an additional 22 and 33 patients, respectively, were enrolled and received 1.1 × 10^14^ vgs/kg i.v.^260,261^ The mean age at dosing was comparable in both studies (3.7 vs. 4.1 months). The US study had co-primary endpoints of unassisted sitting for at least 30 s and ventilator-free survival, while the EU study defined the primary endpoint as unassisted sitting for at least 10 s, and ventilator-free survival as the secondary endpoint. Both studies administered a prednisolone regimen. In STR1VE, 13/22 patients achieved independent sitting for at least 30 s, and in STR1VE-EU, 14/20 patients achieved sitting independently for at least 10 s, with one reaching 30 s. Of note, the EU trial had broader inclusion criteria, with some patients showing signs of more advanced disease, limiting the direct comparison of both studies. In addition, both trials reported elevated liver enzymes and thrombocytopenia after post-infusion.^260,261^

Long-term follow-up results from the initial START trial were reported in 2021.^262^ The longest follow-up was 6.2 years, and patients retained motor skills gained during the initial trial. Encouragingly, two patients acquired new motor milestones and could stand with assistance. The safety profile remained very good, with no TEAEs reported.^262^

The availability of an approved therapy allows the disease to be included in newborn screening, which could identify pre-symptomatic patients. This raises the possibility of prophylactic treatment.

The SPR1NT trial investigated the therapeutic outcome of prophylactic treatment with Onasemnogene abeparvovec (Zolgensma) at 1.1 × 10^14^ vgs/kg in 14 patients with two *SMN2* copies (median age: 21 days) and 15 patients with three *SMN2* copies (median age: 32 days).^263,264^ Prednisolone was given prophylactically and post-treatment. All patients with two *SMN2* copies achieved the primary endpoint of independent sitting for over 30 s. 11/14 patients attained this milestone within the normal developmental window, with similar results observed for independent standing. With the understanding that there may be different definitions of walking, 9–10/14 children were able to walk independently, with 4–5 achieving this within the expected developmental window. Importantly, all patients remained free of permanent ventilation and alive at the end of the study. Elevated liver enzymes, cardiac serum markers, thrombocytopenia but no thrombotic microangiopathy (TMA), and transient areflexia/hyporeflexia were reported, which resolved in all but one patient and were classified as unrelated to the treatment.

Patients with three *SMN2* copies manifest milder disease, which was reflected in independent standing for at least 3 s and walking for at least five steps in 14/15 children. 14/15 achieved standing and 11/15 achieved walking within the expected developmental window. Similar to the two *SMN2* copy groups, all patients with three *SMN2* copies remained alive and free from permanent ventilation at the end of the study. Reported TEAEs were comparable to the two *SMN2* copies group.

The results in both cohorts emphasize the benefit of early pre-symptomatic treatment, which resulted in degrees of clinical improvement not observed before with Zolgensma. It also highlights the favorable safety profile of the treatment, although there have been reports of more severe hepatotoxicity and even death.^265^ Zolgensma obtained FDA approval in 2019, and as of March 20, 2023, over 3000 patients have been treated with Zolgensma, according to Novartis. Completed or ongoing studies are now exploring new aspects, including combination therapies or transitioning patients from conventional therapy to Zolgensma.^266^

#### Metabolic diseases

Genetic defects causing metabolic disorders can have systemic or organ-specific manifestations, with implications on vector design and route of administration (e.g., lysosomal storage diseases with primary CNS manifestation). Here, Tay Sachs disease (TSD), Sandhoff disease (SD), and Canavan disease (CD) will serve as examples of neurometabolic conditions with currently active clinical trials (NCT04669535, NCT04798235, NCT04833907, and NCT04998396).

TSD and SD are lysosomal storage diseases, categorized as GM2 gangliosidoses resulting from autosomal recessive, loss-of-function mutations in the ubiquitously expressed gene encoding β-N-acetylhexosaminidase (*HEX*). HEX forms a dimer comprised of subunits α and β (HEXA), β and β (HEXB), or α and α (HEXS). TSD and SD are caused by mutations in the genes encoding the α- and β-subunits, respectively.^267^ Clinically, both diseases present in infantile, juvenile, and adult forms, which differ in onset and severity.^268–270^

One challenge is the need for a stochiometric balance of the α- and the β-subunits for optimal functionality of the HEX dimer. With rAAV’s limited packaging capacity, one strategy is simultaneous delivery of the α- and β-subunit with two separate vectors. Alternatively, a bicistronic rAAV has been developed to express the α- and β-subunits together.^271,272^

In 2022, an expanded access trial in two TSD patients demonstrated the safety of intrathecal (i.t.) infusion of two rAAV.rh8 vectors expressing α and β subunits. The patients were 30 months (1.0 × 10^14^ vgs; i.t.) and 7 months (4.05 × 10^13^ vgs; combined bilateral intrathalamic and i.t.) old and received immune suppression with rituximab, sirolimus, and prednisolone. The procedure and vector were well-tolerated. Although the functional outcome was not part of the study’s endpoints, both patients showed signs of disease stabilization. This first human TSD rAAV gene therapy study was a milestone in evaluating intrathalamic delivery and has paved the way for an ongoing clinical trial.^273^

Currently, two clinical trials are active in Canada (NCT04798235) and the US (NCT04669535). The Canadian phase I/II trial utilizes rAAV9 to deliver the *HEXA* and *HEXB* transgene with one vector for infantile TSD or SD patients via a single i.t. dose. In contrast, the trial in the US is designed as a dose escalation study of four different vector doses administered via bilateral intrathalamic route and i.t./cisterna magna combined. The study uses two rAAV.rh8 vectors delivering the *HEXA* or *HEXB* transgene. Results from these trials are pending.

CD is an autosomal recessive inherited progressive neurodegenerative disease hallmarked by spongiform degeneration and elevation of N-acetylaspartate (NAA). Loss of function mutations in the enzyme *ASPA* causes accumulation of NAA in the CNS and urine. Affected children typically never learn to walk, talk, control the movement of their head, or gain any level of independence.^274,275^ Many patients succumb to the disease within the first years of life. A clinical trial evaluated the safety of intracranial rAAV administration for the first time in the treatment of CD.^276^ After this clinical trial, studies to optimize vector design could achieve varying degrees of age-dependent prevention and reversal of the disease in mice.^132,277–280^ It is likely that differences in vector design and route of administration might account for the variable outcomes reported in preclinical studies.

Combining intracerebroventricular (i.c.v.) and i.v. routes of administration were employed to reduce the overall vector dose in an early single-patient trial.^281^ However, comparative studies are needed to evaluate this strategy.

Currently, there are two clinical trials ongoing, with one actively enrolling at the time of writing (NCT04833907 and NCT04998396). The two trials use different vector designs and routes of administration (intracranial delivery vs. i.v. infusion). This difference in route of administration necessitates differences in dose. Data for both trials have been presented at scientific conferences and look to be promising. With both trials ongoing, direct comparison is not feasible at the time of this writing.

#### Hematological diseases

Non-oncologic hematologic conditions can be classified into cellular or acellular diseases, meaning that genetic mutations affect cellular function (e.g., sickle cell disease)^282^ or components of blood (e.g., hemophilia),^283^ respectively. The distinction is crucial for gene therapy as it determines the target cell/organ.

In cellular hematologic conditions, the target is a cellular blood component, typically bone marrow. Blood cells have a short half-life, so directly targeting them by rAAV loses therapeutic efficacy quickly as the cells turn over. In contrast, bone marrow stem cells constantly divide to produce new cells, which dilutes the rAAV genome. Nevertheless, this may be a desirable effect if the host genome can be edited or if the gene therapy-expressing cells gain a competitive advantage. Acellular components, in contrast, are released by cells into the bloodstream. This provides an opportunity to express the transgene in any cell type that can export proteins into the bloodstream.

Hemophilia A and B are prime examples of hematologic disorders caused by a deficiency in secreted acellular blood components.^283^ The estimated prevalence for hemophilia A and B are ~12:100,000 and 4:100,000, respectively.^284^ Hemophilia A and B are caused by factor VIII (FVIII) and factor XI (FXI) deficiency, respectively, and both are recessive X-linked conditions.^285^ FVIII is synthesized in endothelial cells, whereas FIX is produced in hepatocytes.^286–288^

Hemophilia A and B patients have a substantially increased risk of bleeding spontaneously or with minor trauma, causing high morbidity and mortality (e.g., intracranial hemorrhage).^289,290^ In addition, repeated bleeding causes local reactions and disabling cellular degeneration (e.g., hemophilic arthropathy).^291^ Notably, patients have reduced overall survival compared to the general population.^290^

Prior to gene therapy, treatment of severe hemophilia A or B required repeated administration of the deficient factor or drug to modulate the coagulation cascade.^292–294^ Healthcare spending for those drugs has been rising, reaching ~$1.5 billion in the US in 2019.^295^ Gene therapy emerges as an ideal modality due to its potentially enduring effect and the clinical knowledge (i.e., natural history) in diagnosing and treating hemophilia, a critical aspect in gene therapy development that is often missing in ultra-rare conditions. In addition, FVIII and FIX are secreted, which means ectopic expression in a tissue could produce the factor (e.g., muscle). Hemophilia severity is classified based on plasma FVIII or FIX activity: severe <1%, moderate 1–5%, and normal 5–40%.^285^ This provides clear target levels to achieve partial (>1%) or complete (>5%) disease correction. Such meaningful thresholds rarely exist or are known in other diseases.

First attempts to develop gene therapy for hemophilia using viral delivery platforms predate the successes of rAAV.^296,297^ These early non-rAAV studies could not achieve lasting therapeutic factor levels or trigger immune responses against the viral vector.

In 1997, two studies could demonstrate persistent human FIX (*hFIX*) expression using rAAV (rAAV.*hFIX*) via intramuscular (i.m.) or intraportal vein (liver-directed) injection.^298,299^

A possible challenge could arise from FIX activity inhibiting antibodies, which were found after i.m. rAAV.*hFIX*.^300^ However, using species-specific transgenes might ameliorate the formation of such antibodies or other immune responses, as demonstrated in hemophilia B dog studies that utilized canine FIX (*cFIX*).^301,302^ While intraportal delivery is invasive and dogs required FIX transfusion prior to the procedure, slightly higher cFIX plasma levels were observed versus the i.m. study.^301,302^ However, this conclusion is unreliable due to differences in study parameters, including vector dose.

In 2000, a phase I trial using i.m. delivery of rAAV.*hFIX* reported a favorable safety profile,^303^ which was confirmed three years later after the completion of the study. Notably, no anti-FIX inhibitory or non-inhibitory antibodies were detected, attributed to patient selection (only missense mutations) and limiting the per-site total rAAV dose, which was identified in animal models to correlate with the formation of inhibitory anti-FIX antibodies. However, patients remained below the 1% FIX serum level, which is considered the lowest therapeutic goal.^304^ A separate phase I/II trial with rAAV2.*hFIX* was given via hepatic artery delivery and only achieved transient FIX expression, likely due to anti-AAV capsid cellular immune responses causing hepatocyte destruction and transgene loss. This finding spearheaded a major focus on rAAV immunology and its impact on clinical safety and efficacy.^305,306^ To reach therapeutic efficacy, subsequent efforts to increase FIX expression were undertaken by optimizing several parameters, including the use of newly discovered rAAV capsids, transgene codon optimization, promoter selection, route of administration, and immune suppression. Additional innovation came from the discovery of the Padua variant of FIX in 2009, which has supraphysiologic activity.^305^ First, a phase I trial combined i.v. delivered hepatotropic rAAV8 and codon-optimized hFIX transgene resulting in 2–12% FIX activity with no immune suppression being used.^307^ As expected, a humeral as well as T cell-mediated anti-AAV8 capsid immune response was observed. The T cell response correlated with dose generally, but not universally, suggesting patient-specific factors.^307^ The patients from this trial were included in a follow-up study with four new patients in the high-dose group, which demonstrated overall FIX activity of 1–6% over a median of 3.2 years.^308^ While the safety profile was favorable, some patients in the high-dose group experienced delayed elevation of liver enzyme levels associated with decreased FIX activity, which resolved with glucocorticoid administration.^308^

While the change to the rAAV8 capsid and codon-optimization of the transgene showed promising improvements, it did not consistently achieve therapeutic FIX activity. To overcome these challenges, a newly engineered capsid with increased liver tropism and a codon-optimized Padua FIX variant was designed.^309^ Notably, the dose (5.0 × 10^11^ vgs/kg) was 4-fold lower than the high dose in the 2011 and 2014 trials (2.0 × 10^12^ vgs/kg). Despite the reduced dose, the FIX activity reached a mean of 33.7% (range 14–81%).^309,310^ Prophylactic immunosuppression was not administered and only two patients required prednisolone for transient liver enzyme elevation, highlighting the excellent safety profile. Clinically, the annualized bleeding rate decreased significantly from a mean rate of 11.1 to 0.4.^310^ Overall, the increased activity of the Padua variant turned out to be highly beneficial and was utilized in subsequent clinical trials.

The hemophilia B trials uncovered several aspects of rAAV gene therapy in humans that were not anticipated, including a gradual decrease in transgene expression and immune responses. Currently, FDA-approved gene therapy for hemophilia B (i.v. rAAV5 Padua, Hemgenix, Etranacogene dezaparvovec) is on the market, while other rAAV products (verbrinacogene setparvovec (FLT180a) using an rAAVS3 capsid and Fidanacogene elaparvovec, formerly known as SPK-9001 and PF-06838435) are in clinical trials but await FDA approval.

A notable challenge for hemophilia A is the ~7 kb *FVIII* transgene, exceeding rAAV’s packaging capacity^311^ and necessitating the use of a truncated form. The therapeutic efficacy of a truncated FVIII was evaluated in nine patients by i.v. rAAV5 delivering a codon-optimized *FVIII* variant (*FVIIISQ*; missing the B-domain).^312^ The results from different dosing groups (6.0 × 10^12^ vs. 2.0 × 10^13^ vs. 6.0 × 10^13^ vgs/kg) demonstrated that the highest dose achieved therapeutic FVIII activity for the 52-week study period. Encouragingly, only the high-dose patients received immune suppression, and no clinically adverse effects were identified.^312^ However, transgene expression decreased over time.^313^

A separate trial evaluated a rAAV3-derived engineered capsid expressing FVIIISQ at four doses ranging from 5.0 × 10^11^ to 2.0 × 10^12^ vgs/kg in 18 patients.^314^ While it achieved therapeutic benefit, two of the 18 patients lost transgene expression entirely due to an anti-rAAV capsid immune response despite using glucocorticoids. Of note, attempts to wean several patients off glucocorticoids resulted in a cellular immune response. Although vector properties between the trials by Savita et al. ^312^ and George et al. ^314^ might explain the difference in transgene expression and maintenance, it was hypothesized that high expression levels might trigger an unfolded protein response with subsequent FVIII loss.^314^

A follow-up phase III trial using rAAV5hFVIIISQ enrolled 134 patients receiving a single 6.0 × 10^13^ vgs/kg i.v. dose.^315^ This one-time treatment significantly increased FVIII activity and reduced exogenous FVIII use, and the annual bleeding events improved compared to exogenous FVIII prophylaxis. Elevated liver enzymes were found at a median of 8 weeks, which decreased with glucocorticoid treatment in 96.2% of the patients.^315^ The two-year follow-up data showed a similar trend as in the previous phase I/II trial, with decreased annual bleeding rate and exogenous FVIII use. However, decreasing transgene expression over time was observed as well. Unexpectedly, the FVIII levels in the phase III trial were lower than what was found in the phase I/II trial at one- and two-year follow-up.^316^ This therapeutic vector recently obtained FDA approval (roctavian, Valoctocogene Roxaparvove, rAAV5hFVIIISQ), and it will be vital to conduct follow-up clinical studies to determine its durability.

#### Neuromuscular diseases

Neuromuscular diseases comprise conditions affecting neurons and muscle cells,^317^ with muscular dystrophies primarily affecting muscle cells. DMD is the most common dystrophy,^318^ and the FDA recently granted approval for an rAAV-based gene therapy.^319^ DMD is a rare X-linked and progressive disease with a prevalence of ~15.9–19.5/100,000,^320^ which primarily affects males, although rare cases in females have been reported.^321^ It is caused by mutations in the dystrophin gene resulting in absent or dysfunctional dystrophin protein. It manifests with muscle weakness, loss of the ability to walk, respiratory impairment, and cardiomyopathy,^322–324^ with the latter two commonly being the cause of patient mortality.

The dystrophin gene consists of eight promoters and 79 exons to generate a ~11.4 kb cDNA and 427 kDa muscle isoform protein.^321^ Mutations typically lead to a truncated protein in DMD patients.^321^ The large dystrophin cDNA size poses a major challenge to rAAV-based gene replacement therapy because it exceeds the packaging capacity, so strategies have focused on identifying the minimally required protein sequences. Two different dystrophin transgenes have been developed: ~6–8 kb mini-dystrophin and ~4 kb microdystrophin.^325–328^ The mini-dystrophin needs to be divided and delivered by two rAAVs, while the microdystrophin fits into one rAAV.^325^ Other strategies using rAAV involve exon skipping,^329^ *GALGT2* delivery^330^ (shown to prevent muscle pathology in mice), and gene editing to correct the dystrophin gene or activate non-muscle dystrophin isoforms using CRISPR.^331,332^ The focus here is on strategies that have been translated to human application.

In 2006, a trial evaluated a hybrid rAAV2.5 expressing miniature dystrophin by i.m. injection in six patients, aged 5–11 years.^333^ Patients were divided into two dosing groups: 2 × 10^10^ and 1.0 × 10^11^ vgs/kg. While some muscle fibers were found to be mini-dystrophin-positive in 2/6 patients at day 42 post-treatment, the failure to find dystrophin-positive cells at later time points might point towards an immune-mediated process. This is also supported by findings of transgene-specific T cells. Surprisingly, 2/6 patients were positive for T cells against a domain of the mini-dystrophin gene prior to receiving gene therapy, suggesting that the mutated endogenous dystrophin might not be sufficient to induce immune tolerance.^333^

To achieve broad targeting of affected muscle cells throughout the body, a trial evaluated the safety and therapeutic efficacy of i.v. infusion of 2.0 × 10^14^ vgs/kg rAAV.rh74.micro-dystrophin in four patients with a mean age of 4.8 years.^334^ Encouragingly, all patients in this phase I/II trial improved clinically and histopathologically, which correlated with age and disease severity at the time of treatment. In contrast to the earlier trial results from 2010, T-cell activity against the micro-dystrophin protein was not detected. Liver enzyme levels rose after rAAV infusion but remained moderate and normalized with corticosteroid treatment. No signs of TMA were found.^334^ A randomized, double-blinded, placebo-controlled clinical trial (NCT03769116, delandistrogene moxeparvovec) involving 41 patients was just completed. Notably, this therapy obtained FDA approval in 2023.^319^

An alternative strategy to gene replacement therapy involves the use of disease-modifying genes. In 2022, a first-in-human trial treated two patients (6.9 and 8.9 years of age) with rAAV.rh47 expressing *GALGT2* at 5.0 × 10^13^ and 2.5 × 10^13^ vgs/kg per leg via bilateral femoral vein isolated limb perfusion, respectively.^335^ Isolated limb perfusion has been widely evaluated in animals as an attempt to increase muscle transduction but also reduce the total dose. GALGT2 acts as a β1,4 N-acetylgalactosaminyltransferase in muscle glycosylating α-dystroglycan and has shown positive effects on muscular pathology. No relevant liver toxicity was reported, despite the detection of antibodies against the rAAV capsid and mild T-cell activity against rAAV and the GALGT2 protein. While the patient number was too small to draw significant conclusions, it was noted that the younger patients receiving the higher dose showed greater improvement.^335^

Other ongoing trials are still active but not recruiting, including a randomized phase I/II trial by Solid Bioscience (NCT03368742) and a non-randomized phase Ib trial by Pfizer (NCT03362502), which both utilize rAAV9 to express truncated dystrophin under the control of muscle-specific promoters. These trials evaluate increasing i.v. doses and include children and adolescents but exclude patients with advanced degrees of cardiac and respiratory impairment.^323^

Defining eligibility criteria can be particularly challenging and highlights current gaps in understanding and predicting the interaction between rAAV gene therapy and the disease. A recent single-patient DMD trial using i.v. rAAV9 at 1.0 × 10^14^ vgs/kg expressing dCas9 linked to VP64 to activate expression of cortical dystrophin ended fatally.^336^ The patient, who was non-ambulatory and had advanced disease at the time of dosing, developed worsening cardiac function on day five and acute respiratory distress syndrome on day six post-dosing, resulting in cardiopulmonary arrest and death on day eight post-treatment. The concluding assessment led to the hypothesis that the patient developed cytokine-mediated capillary leak syndrome, as tests for the humeral or cellular immune response against the rAAV capsid or transgene were negative. There were also no signs of TMA, as has been seen in high-dose DMD trials. A second patient, who was part of the Pfizer trial (NCT03362502), died six days after receiving 2.0 × 10^14^ vgs/kg of rAAV9. The patient was younger and received double the dose as in the single-patient trial above. Although no autopsy was performed, the current hypothesis centers on an innate immune response in the cardiac muscle causing heart failure (HF).^337^

#### Cardiovascular diseases

HF is a syndrome defined by structural or functional alterations of ventricular filling or emptying.^338^ It is highly prevalent with ~6 million people affected in the US.^339^ Calcium is crucial in cardiac function and is also a target for conventional drug development. One preclinically successful strategy is the overexpression of ATPase Sarcoplasmic/Endoplasmic Reticulum Ca^2+^ Transporting 2 (SERCA2a), which pumps calcium into the sarcoplasmic reticulum (SR) during the cardiac relaxation process.^340,341^

The CUPID trial evaluated overexpressing *SERCA2a* using rAAV1 (rAAV1.*SERCA2a*).^342,343^ It was designed as a phase I/II trial, with the phase I portion being an open-label sequential dose escalation study^342^ and phase II being randomized, double-blind, and placebo-controlled.^343^ During the phase I study, a single intracoronary administration for heart-directed rAAV1.*SERAC2a* delivery was evaluated at 1.4 × 10^11^, 6.0 × 10^11^, and 3.0 × 10^13^ vgs (*n* = 3 patients per cohort). All treated patients had New York Heart Association (NYHA) Class III HF at the time of treatment. Some patients displayed symptomatic improvement and tolerated the treatment well. One patient died, which was determined to be unrelated to the treatment. Overall, the favorable safety profile encouraged the transition to the phase II portion of the study, which enrolled 25 patients receiving treatment and 14 receiving placebos. Doses were assigned to three cohorts, 6.0 × 10^11^ (*n* = 8), 3.0 × 10^12^ (*n* = 8), and 1.0 × 10^13^ vgs (*n* = 9).^343^ All patients had NYHA Class III HF at the beginning of the phase II study. Overall, a trend of improvement in the treated patients versus placebo was observed; however, most outcome criteria did not reach statistical significance, except for left ventricular end-diastolic volume. The study confirmed the favorable safety profile observed in phase I.^343^

The three-year follow-up results of the CUPID phase II could not demonstrate significant improvement in survival. However, a cumulative analysis for non-terminal events reached statistical significance encouraging CUPID 2.^344^ CUPID 2 assigned a total of 250 patients to the treatment (*n* = 123) or the placebo group (*n* = 127).^345^ Similar to prior trials, critical outcome measures did not show significant differences (e.g., survival). Interestingly, early terminal events were significantly increased in the intervention group.^345^

Other studies investigated the benefit of rAAV1.*SERCA2a* on left ventricular end-diastolic volume (AGENT-HF) or safety in patients with concomitant left ventricular assist device (LVAD) (SERCA-LVAD) and the impact of circulating NAbs.^346,347^ Both trials were terminated after discouraging CUPID 2 trial results emerged.

Although these trials did not result in clinically significant improvement in outcome, they highlight the potential of rAAV gene therapy for cardiac diseases. New, promising cardiac gene therapies using rAAV are being developed, including using rAAV9 to express *LAMP2B* for the treatment of Danon disease, an X-linked disorder causing severe cardiomyopathy. A phase I (NCT03882437) trial has completed recruitment, and the interim results suggest a favorable safety profile and clinical improvement, with occurrences of TMA and acute kidney injury being resolved by treatment.^348,349^ A corresponding phase II (NCT06092034) study is actively enrolling males of at least 8 years of age as a single-arm study for administration of a single i.v. infusion. The trial is expected to be completed in 2025.

#### Cancer

rAAV has shown promise in preclinical studies for cancer, but its translation to use in humans has been limited.^350–353^ At the time of this writing, only one trial using rAAV for ex vivo gene therapy in gastric cancer is active (NCT02496273). Thus, this section focuses on preclinical achievements and rAAV-based strategies to treat oncologic conditions. For a more detailed understanding, we refer readers to several in-depth reviews.^354,355^

Anti-tumor strategies using rAAV share similar targets and concepts as conventional anti-tumor regimens. However, gene therapy offers specific advantages, such as modulation or knock-down of gene expression. rAAV transduces dividing and non-dividing cells, enabling broad cell targeting within heterogeneous tumor tissue. This property can be used to deliver cytokine transgenes to tumor cells to recruit immune cells and trigger an anti-tumor immune response.

A proof-of-concept study showed that rAAV-mediated interferon β (IFNβ) expression suppressed colorectal cancer cells in a xenograft mouse model.^356^ In a non-randomized, non-blinded, retrospective human study, the addition of IFNβ to temozolomide therapy extended survival in glioblastoma (GBM) patients.^357^ Driving expression of IFNβ by rAAV-transduced (rAAV.*IFNβ*) tumor cells can overcome IFNβ’s short half-life and boost therapeutic efficacy, as evidenced in an invasive orthotopic xenograft GBM mouse model, where intracranial delivery almost doubled overall survival.^358^

Taking advantage of rAAV’s adaptable capsid, tumor-directed capsid engineering can increase transduction efficiency and specificity while mitigating possible adverse effects. Indeed, rAAV displaying a Her2 ligand showed increased transduction specificity.^359^ The expression of HSV thymidine kinase converts ganciclovir into a toxic product which is subsequently incorporated into the tumor DNA to induce cell death. Delivery of this gene reduced tumor burden and extended survival compared to an animal group that received the approved anti-cancer drug Herceptin.^360^

While engineering capsids to deliver cancer therapeutics offers high specificity, this approach is time-consuming and labor-intensive. An alternative approach is to screen existing rAAV capsid libraries for tropism after delivery by different routes of administration to determine the best possible combination. For example, rAAV7 and nine have been found to achieve high transduction of prostate tissue in mice.^361^ This enhanced transduction of prostate tissue can be used to knock down miRNAs associated with prostate cancer, which has been found to slow disease progression in a mouse prostate cancer model.^362,363^

These proof-of-concept studies highlight promising anti-cancer strategies using rAAV. However, critical questions must be addressed before human application is feasible. There are concerns regarding the safety of sustained therapeutic transgene expression, particularly after tumor elimination or relapse. Similar to conventional chemotherapy, recurrent tumors might be resistant to or able to escape the therapy, making ongoing transgene expression ineffective. Given that cytokine therapies often have substantial side effects, unregulated transgene expression could cause additional complications. Improved regulated gene therapy strategies need to be developed together with advances in understanding of tumor biology.

### Challenges and limitations in translating rAAV-based gene therapies to clinical practice

rAAV capsid performance is tightly connected to the route of administration, prompting exploration of different delivery routes, including intravascular, direct intra-tissue injection, and delivery into pre-existing body cavities or fluid space.^364–371^ Each route and capsid selection must be determined in the context of the disease, target area, organ system, and other factors such as patient age (Table 2).

**Table 2:** Pros and cons of different routes of administration of rAAV

Routes of administration	Pros	Cons	Examples
Intravascular	• Widespread transduction	• Transduction of non-target organs	i.v., i.a.
	• Reach most organs	• Potentially high doses required	
Intra-tissue	• Localized delivery	• Invasive	i.m., i.p.
		• Decreased spread	
		• Possible local reaction	
Intra-cavity	• Spread in preformed space	• Transduction potentially limited to preformed space	s.p., s.r., i.v.t
		• Potentially uneven distribution	
Intra-fluid space	• Utilization of fluid dynamics to distribute vector	• Long vector travel distance	i.c.v., c.m., i.t.

*i.v.* intravenous, *i.a.* intra-arterial, *i.c.v.* intracerebroventricular, *i.m.* intramuscular, *i.t.* intrathecal delivery, *c.m.* cisterna magna, *i.p.* intraparenchymal, *s.p.* subpial space, *s.r.* subretinal, *i.v.t.* intravitreal

A classic example is the BBB protecting the CNS, which presents a barrier that gene therapies need to overcome. Some rAAV capsids efficiently cross the BBB.^364–366,372^ Targeting the CNS via systemic i.v. delivery is not optimal if the therapeutic target is merely a small brain area, as the required vector dose and possible side effects would be high without achieving superior transduction of the target area. In such cases, direct and localized delivery is critical, as exemplified in AADC.^249,251^ However, even within a confined delivery area, capsid selection must be scrutinized. rAAV9 displays substantial tissue spread and relatively broad tropism even with localized delivery, while serotypes such as rAAV2 have a more limited transduction capacity.

Another challenge is the translation from preclinical to clinical studies. To mitigate vector costs and risks from high doses as used in i.v. delivery, intra-CSF space administration is widely utilized for widespread CNS transduction.^373–375^ The challenge of extrapolating findings in mouse, rat, or even larger animal brains to humans is daunting. For example, rAAV9 can achieve global brain transduction in mice, where the distance between the lateral ventricle and cortex is merely a few millimeters. In humans, this distance spans several centimeters. Thus, clinical trials using CSF delivery in humans for widespread CNS transduction have to demonstrate the translatability of this delivery mode.

### Immunogenicity of rAAV

**Fig. 6:**
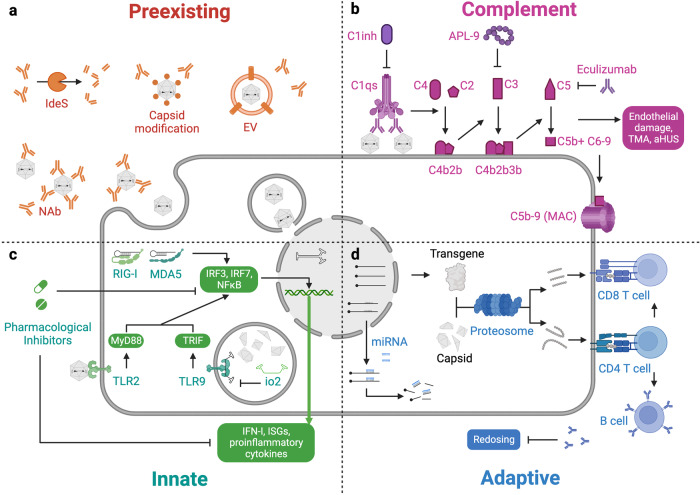
Immune responses in rAAV-based gene therapy. **a** Pre-existing AAV-specific antibodies can interact with rAAV and block its target cell entry. Bacteria-derived endopeptidase IdeS and its homologs can cleave the intact antibody into Fab and Fc fragments, thus reducing its half-life in circulation and eliminating Fc-mediated functions. Alternatively, the capsid can be modified or encapsulated by EVs to prevent antibody recognition. **b** Complement activation is observed when high doses of rAAV are administered. Though the exact mechanism remains unclear, a leading hypothesis is that rAAV adhering to the cell surface activates the classical pathway by binding to complement C1qs, which cleaves C4 and C2 to form the C3 convertase C4b2b. C3 is then cleaved and forms C5 convertase that cleaves C5 into C5b, which associates with C6–9 to form the membrane attack complex (MAC) that directly causes cell lysis, resulting in liver or kidney injury. Complement activation mediates endothelial damage, thrombotic microangiopathy (TMA), and atypical hemolytic uremic syndrome (aHUS). Cyclic peptide APL-9 is a C1 inhibitor (C1inh) and eculizumab is an inhibitor of C1, C3, and C5, which may suppress the complement activation cascade. **c** Innate receptors, including TLR2 for virus capsid, TLR9 for unmethylated CpG, and RIG-I/MDA5 for dsRNA, have been shown to promote inflammation. Through a series of signal transduction events, IRF3, IRF7, and NF-κB will become phosphorylated and translocate to the nucleus, where they activate type I interferons (IFN-I), interferon-stimulated genes (ISGs), and proinflammatory cytokines that mediate antiviral immunity and propagate inflammation. Many pathway components can be targeted by pharmacological inhibitors. In addition, the telomere-derived io2 sequence can suppress TLR9-mediated recognition of the transgene. **d** Transgene and capsid can be processed by the proteasome into peptides, which are presented by MHC-I or MHC-II molecules. CD8 and CD4 T cells that recognize those peptides will become activated. CD8 T cells may directly kill the rAAV-transduced cell, while CD4 T cells can help both CD8 T cells and B cells to enhance their effector function. B cells with specificity to the capsid can release antibodies that eliminate future possibilities of redosing. miRNA-binding sites engineered to the transgene can interact with cellular miRNA, leading to transcript degradation. In antigen-presenting cells, this design may prevent the presentation of transgene-derived peptides to T cells. In addition, anti-CD20 antibody rituximab and mTOR inhibitor rapamycin can be used to reduce adaptive responses. Figure created with Biorender.com

In addition to physical barriers, rAAV encounters numerous biological barriers instituted by the immune system, including pre-existing immunity, complement activation, innate pattern and danger receptors, and adaptive B cell and T cell immunity (Fig. 6). Here, we discuss how each immunological barrier negatively impacts vector transduction and what potential solutions are available.

#### Pre-existing immunity

Pre-existing humoral response against the rAAV capsid, measured as rAAV-specific NAbs, likely comes from the immunological memory of past encounters with wtAAV. Newborns harbor maternal AAV NAbs for the first 6 months.^56^ After that, the neutralization titer drops but reappears from 7–9 months old onward. The overall seropositive rate ranges from below 10% to over 90%, depending on the serotype and geographical location.^376^ This is the single most detrimental burden to rAAV gene therapy because even low levels of seropositivity can markedly reduce transgene expression if the vector is given via i.v. administration.^377^

During active engagement with the foreign antigen, some IgG-expressing B cells will differentiate into long-lived plasma cells that primarily reside in the bone marrow and continue to express antigen-specific IgG long after the resolution of infection. While IgM levels tend to drop sharply after the resolution of the infection, IgG levels can be maintained for a substantially longer time and constitute the main humoral barrier for productive infection.^378^

NAbs also present the most significant challenge in redosing. Because rAAV genomes are typically maintained as circularized episomes,^379^ their average number per cell reduces by a factor of two every time the host cell divides and is lost when the host cell dies. Hence, subsequent doses of gene therapy may be required during a patient’s lifetime,^380^, especially for gene therapies that target organs with active cell turnover, such as the liver. However, redosing is inhibited by NAbs developed in response to the first dose.

##### rAAV capsid–antibody interactions especially for gene therapies

The antibody footprint of the complementarity-determining region (i.e., where the antibodies touch the antigen) on the AAV capsid surface can be captured through cryo-EM or peptide scanning.^21^ Some antigenic “hot” areas have been identified, such as the icosahedral 3-fold axes, the 5-fold axes, and the 2/5-fold wall.^381^ A recent investigation in patients receiving Zolgensma found that ~75% of antibodies are bound to the 2-fold surface.^382^ While these discoveries help guide capsid engineering toward escape from pre-existing immunity imposed by antibodies,^70^ it is important to keep in mind that the rAAV is quite small relative to the antibody, such that a few antibodies binding to the virion is sufficient to cover the viral surface and block its cell entry.

##### Impact on local vs. systemic routes of administration

Intravenous infusion is most susceptible to rAAV-specific circulating antibodies.^383^ For local injection routes into immune-privileged organs, such as the eye and the CNS, the restriction imposed by rAAV-specific antibodies is less severe and there is a possibility of redosing.^384^ While pre-existing NAbs are much lower in the vitreous humor than in the serum, the NAb level in the vitreous humor is negatively associated with the integrity of the BBB,^385^ an important indicator of potential effects of ocular gene therapy. In NHPs receiving ADVM-022 via the intravitreal route, injection in one eye resulted in NAbs in the serum and the injected eye, but not in the uninjected eye.^386^ Intravitreal injection of the remaining eye 2 months later did not lead to exacerbated inflammation. The transgene expression in the second eye trended lower compared to the first eye but was within the predicted range.

Interestingly, this differential susceptibility to circulating NAbs may be explored as a strategy to limit vector transduction in off-target tissues.^387^ When performing intrathalamic or i.c.v. injection, the presence of NAbs blocked transgene delivery to the liver but not to the CNS.^384,387^

##### Screening for pre-existing immunity

The level of rAAV capsid-specific total antibody (TAb) level can be measured using enzyme-linked immunosorbent assays. To determine patient eligibility for clinical trial participation, many trials employ a cell-based transduction inhibition (TI) assay to measure how dilute the patient’s serum needs to be to allow optimal rAAV transduction.^307,388,389^ There are instances when the serum is TAb-positive and TI-negative, indicating that not all AAV-specific antibodies inhibit transduction. More intriguingly, some sera are TAb-negative and TI-positive, suggesting the presence of inhibitory serum components that are not antibodies.

##### Evasion strategies of pre-existing immunity

CD20 is a highly expressed B cell-specific molecule that can be targeted by rituximab, which is given in several gene therapy trials in conjunction with other immune suppressors, such as steroids and rapamycin, to reduce general inflammation as well as B cell- and T cell-mediated immunity.^390–392^ However, rituximab is not effective at preventing pre-existing humoral immunity because long-lived plasma cells do not express CD20.^383^

rAAV packaged in extracellular vesicles (EVs) may allow partial escape from NAbs.^393–396^ The EVs can be coated with immunosuppressive molecules, such as CTLA-4, for further immune suppression.^397^ Packing rAAVs inside EVs may modify the tissue tropism, which may require additional modifications of the EV similar to modifying the rAAV capsid as discussed above, represents another popular approach for evading pre-existing immunity.

Recently, bacterial endopeptidases that cleave human antibodies have been explored.^398^ Imilifadase (IdeS), an IgG-degrading enzyme from *Streptococcus pyogenes*, has been shown to deplete total IgGs and allowed significantly better vector transduction in rAAV-seropositive NHPs.^399^ Its homolog IdeZ from *S. equi* ssp. *zooepidemicus* demonstrated similar effects.^400^ IdeXork, a proprietary IgG-degrading enzyme, is entering an rAAV-based clinical trial for Late-Onset Pompe disease.^401^ Across multiple studies, it appears that IdeS-type enzymes can provide an IgG-free window of 5–7 days.^399,402,403^ One potential caveat is that IdeS seems to function best when the neutralization titer is only moderately high (~1:108 for AAV3B in NHP).^402^ In one NHP starting with a neutralization titer of 1:696, IdeS reduced the titer to 1:88, which still inhibited transgene expression. Also, an adaptive immune response against IdeS may negate its efficacy. Drawing from transplantation studies, using IdeS the second time may only allow a therapeutic window of 24 h due to the generation of anti-IdeS antibodies.^404–406^

There are also IgM-cleaving enzymes (IceM) from *S. suis* and IceMG, which are engineered from IceM and cleaves both IgM and IgG.^407,408^ In addition to cleaving free-form IgM/G in the serum, these enzymes can cleave IgM from the B cell surface and block complement activation mediated by rAAV9 in vitro.^408^ Whether these will translate to clinical benefits remains to be studied.

#### Complement activation

The complement system consists of a series of highly abundant serum proteins and their associated cellular receptors.^409,410^ Many patients receiving high dose (~1.0 × 10^14^ vgs/kg) rAAV gene therapy developed TEAEs related to complement activation, including thrombocytopenia, hemolytic anemia, TMA, liver enzyme elevation, acute liver injury, and hemolytic uraemic syndrome (aHUS)-like kidney injury.^411,412^ Fatality in association with complement activation has been documented.^413^ It should be noted that as of Jan 13, 2022, nine cases of TMA were recorded out of over 1400 patients (~0.6%) receiving Zolgensma (https://www.fda.gov/media/126109/download).

##### Complement activation mechanism

Complement activation may happen through one of three pathways: classical (IgM/IgG, C1q, and C4), alternative (C3 spontaneous hydrolysis), and lectin (mannose-binding lectin).^409^ All three pathways converge to the formation of C3 convertase, C5 convertase, and finally, the membrane-attack complex that kills cells.^409^

The mechanism of complement activation during rAAV infusion is yet to be elucidated. AAV2 capsid could directly immunoprecipitate C3 and its cleavage products C3b and iC3b,^414^ but rAAV particles at a dose equivalent of 1 × 10^14^ vgs/kg in vitro did not activate C3 in pooled human serum without IgG. Ex vivo experiments demonstrated that AAV in whole blood activated complements only when anti-AAV antibodies were present.^415^ Multiple clinical trials noted the decrease of complement C4 in a subset of rAAV recipients.^416,417^ In addition, a recent study on patients receiving Zolgensma demonstrated that patients who received steroids alone exhibited higher incidences of TMA and higher AAV9-specific IgM and IgG titers, while patients who received steroids together with rituximab and rapamycin presented lower antibody titers and minimal complement activation.^418^ The collective evidence seems to suggest that complement activation in rAAV infusion occurs through the classical pathway. However, two observations reveal unresolved conflicts with this hypothesis. First, lower C4 levels were recorded before the development of rAAV-specific IgM and IgG in an AAV-naïve patient. Second, patients are pre-screened to be seronegative for the specific AAV serotype.

After rAAV administration, neither mice nor NHPs seem to share complement activation features observed in humans. In NHPs, complement activation was transiently detected at day 3 after dosing, even before the detection of AAV-specific IgM, and presented as an increase in Bb and sC5b-9 but not C4b.^419^ In mice, our unpublished results indicate that complement activation is very mild if AAV is given at 2.5 × 10^14^ vgs/kg dose equivalent. A recent study in mice demonstrated that C3b in serum and C5b-9 staining in the liver became detectable at 3.0 × 10^14^ vgs/kg, and at a supra-clinical dose of 7.0 × 10^14^ vgs/kg, mild thrombocytopenia could be detected with the peak of complement activation occurring at day 3.^420^

##### Complement suppression strategies

Several complement inhibitors are being tested for use in rAAV gene therapy, including the C5-blocking monoclonal antibody eculizumab.^412,413^ C3-inhibiting peptide APL-9^421^ and a naturally occurring serum protein C1 esterase inhibitor (C1-inh).^422^

Eculizumab is already used for aHUS and has been given to gene therapy recipients following TMA.^413^ While most patients recover from TMA, one patient eventually dies of acute kidney injury due to new-onset *Staphylococcus epidermidis* sepsis. Eculizumab lowered serum sC5b-9 and slightly increased platelet count but did not rescue the patient, as complements are essential in defense against bacteria.

Because C3 interaction with rAAV capsid promoted virus uptake in mouse macrophages,^414^ complement inhibition may affect rAAV transduction efficiency. This was shown in an ex vivo experiment as APL-9 lowered rAAV uptake by human blood leukocytes in NAb-high serum but not NAb-low serum.^421^ C1-inh, however, increased liver transduction by ~33% and ~28% in non-immunized and immunized mice, respectively.

#### Innate immunity

Myriad cell surface, endosomal, and cytosolic receptors detect molecular signatures commonly associated with pathogens to act as an alert system for the cells’ defense mechanisms and initiate an inflammatory response. Activation of innate sensors typically happens within minutes or hours. Because rAAV is virus-based, some of its inherent features are likely to be detected by these innate receptors. The secretion of cytokines triggered by the immune response has been shown to markedly reduce transgene expression and promote adaptive responses.^423,424^

##### Innate immune receptors shown to interact with rAAV

Innate receptors that have been shown to interact with rAAV include TLR-2, TLR-9, MDA5, and RIG-I.^425^ TLR-2 is a cell surface receptor that can recognize a variety of ligands associated with microorganisms, such as lipoproteins, peptidoglycans, and glycolipids.^426^ It can further expand its ligand recognition repertoire by forming heterodimers with TLR-1 and TLR-6. rAAV2 and rAAV8 both activate NF-κB-mediated cytokine expression in human liver cells in a TLR-2-dependent manner.^427^ TLR-9 is an endosomal membrane-attached receptor responsible for recognizing unmethylated CpG motifs in microbial DNA. Activation of TLR-9 in rAAV not only results in acute expression of cytokines, such as IL-6 and TNF-α, directly downstream of TLR signaling but also promotes infiltration of cytotoxic CD8^+^ T cells, leading to the loss of transduced target cells.^112,424,428^ Depletion of CpG motifs from a rAAV8-based vector to treat hemophilia B led to reduced NAb development.^133^ MDA5 and RIG-I are cytosolic sensors of dsRNA. The 3′ ITR region of rAAV may have promoter activity for the synthesis of minus-strand RNA, which can anneal to the plus-strand transcripts to form double-strand RNA.^423^ Both human hepatocytes and primate retina expressed high levels of MDA5 and RIG-I, which mediated IFN-β production following rAAV infection in vitro.^423,429^

A recent study demonstrated that IL-1 receptor (IL-1R)-MyD88 signaling are critical component involved in rAAV detection, as IL-1R^−/−^ and MyD88^−/−^ mice did not show a transgene-specific T cell response following rAAV8.OVA injection.^430^ The ligands for IL-1R are IL-1α and IL-1β, which are activated following inflammasome activation,^431,432^ while MyD88 acts downstream of TLRs and IL-1R.^433^ Intriguingly, mice deficient in TLR2/3/4/9, NLRP1/3, AIM2, or Caspase-1 exhibited normal or only slightly reduced transgene-specific T cell responses,^430^ suggesting that the rAAV was detected by a yet to be identified innate immune sensor.

##### Strategies to mitigate innate receptor signaling

To reduce TLR-9 signaling, CpG motifs in the rAAV cargo can be removed by modifying codons.^105^ One concern with this approach is that there are differences in the abundance of tRNA molecules, so altering their usage could potentially change the protein folding dynamic and increase the chance of misfolding.^434^ Therefore, incorporating a methyltransferase during rAAV production may be a good alternative.^434^ Another possibility is to incorporate TLR-9 inhibitory sequences. A mammalian telomere-derived ODN TTAGGG repeat sequence can form G-tetrads which can bind to TLR-9 in order to inhibit its dimerization and signaling.^435^ rAAV with this sequence demonstrated reduced immunostimulatory capacity, leading to less T cell infiltration in liver, muscle, and retina across various animal models.^436^

Pharmacological inhibitors of innate signaling pathways are another option. The blockade of IL-1 (inflammasome cytokine) reduced the frequency of transgene-specific CD8^+^ T cells,^437^ while inhibiting IL-6 (TLR cytokine) resulted in reduced antibody responses against the rAAV capsid.^438^ Inhibiting IRAK4 (TLR kinase) led to lower TLR-9 cytokine expression in vitro, and in vivo testing is underway.^439^

#### Adaptive immunity

Adaptive immunity refers to the antigen-specific B cell and T cell responses. B cells can produce antibodies targeting the rAAV capsid, which eliminates the possibility of redosing. Cytotoxic CD8^+^ T cells can recognize foreign peptides from transgenes and the rAAV capsid, and initiate the killing of transduced cells.^306,440,441^ CD4^+^ T cells can help B cell and cytotoxic T cell responses by secreting Th1, Th2, or Th17-type cytokines.^441^ The formation of adaptive B cell responses follows the same mechanism as pre-existing humoral immunity, so this section will primarily focus on T cell responses.

##### T cell-mediated adverse effects

Dorsal root ganglia (DRG) are clusters of sensory neurons located along the spinal cord that play a crucial role in transmitting sensory information. NHP, rodent, and piglet models have displayed a high incidence of DRG lesions in a dose-dependent manner.^442–444^ Symptoms suggesting DRG toxicity, including sensation changes, pain, and DRG enhancement on magnetic resonance imaging (MRI), were observed in one human patient.^445^ Part of the injury may be transgene-induced, which is thought to be limited in duration and scope, while severe DRG toxicity is associated with the influx of CD8^+^ T cells and is unaffected by steroids (https://www.fda.gov/media/152000/download). Similarly, myocarditis following systemic rAAV transfer in NHPs was attributed to transgene-specific cytotoxic T cell responses due to the presence of cardiac muscle damage at a later onset, high infiltration of CD8^+^ T cells in severe myocarditis, and high transgene specificity identified by enzyme-linked immunosorbent spot assays.^419^

Anti-capsid T-cell responses have also been demonstrated. One hemophilia B patient receiving 2.0 × 10^12^ vgs/kg AAV2-*FIX* displayed no anti-transgene T cell response but demonstrated an anti-AAV2 capsid T cell response. This is possibly associated with this patient’s human leukocyte antigen (HLA)-B*0702 allele, which is predicted to have high-affinity binding with a rAAV2 capsid peptide VPQYGYLTL.^306^ In a different hemophilia B trial using scAAV2/8, none of the six enrolled patients displayed anti-transgene responses.^307^ However, anti-capsid T-cell responses were clearly present in patients receiving doses at or above 6.0 × 10^11^ vgs/kg. In two patients, the level of anti-capsid T cell response seems to correlate with a sudden increase in alanine transaminase levels.

##### Strategies to reduce adaptive immunity

miRNAs are small RNA molecules that can bind to mRNA transcripts and catalyze transcript degradation.^446^ Cells naturally express certain miRNA molecules to regulate mRNA transcript abundance. For example, miR-142 is highly expressed by APCs.^142^ By engineering a miR-142 binding site (BS) into the 3′-UTR of the transgene, its translation can be selectively inhibited in APCs, thus reducing antigen presentation to T cells.^143^ Applying the same principle, miRNA-183-BS, which binds to DRG-enriched miR-183, significantly reduced transgene expression in DRG and lowered DRG toxicity.^443^

Another potential mitigating strategy is to target the peptide processing and MHC presentation pathway.^447^ The transgene and capsid sequences are known, and the patient’s HLA types can be determined, so prediction programs are being developed to analyze potential interactions. If required, the transgene and capsid sequences can be redesigned to de-immunize immunodominant peptides when significant interactions are predicted, thereby lowering the likelihood of MHC presentation.

Suppressing innate immune responses can also reduce the intensity of adaptive responses.^430^ mTOR is a kinase acting downstream of multiple cellular receptors.^448^ The mTOR inhibitor rapamycin and its derivatives significantly suppressed adaptive B cell and T cell responses, allowing for better transgene expression and redosing in mouse models.^449,450^ Currently, there is no universal strategy to suppress B-cell and T-cell responses in an antigen-specific manner. Steroids suppress both innate and adaptive immunity and are widely used in rAAV gene therapy.^451^ While studies have demonstrated that steroids can increase transgene persistence, others report that certain anti-transgene T-cell responses do not respond to steroids.^418,452,453^

#### Current immunosuppression drugs

Many trials now use a triple immune suppression regimen that includes steroids, rituximab, and rapamycin.^417^ Nearly all of the drugs currently used or being investigated for the suppression of immune responses in rAAV clinical trials were originally developed for other diseases, such as autoimmune diseases, transplantations, and tumors (Table 3). The physiology between those diseases and rAAV-based gene therapy can be highly divergent, which calls for further drug development specifically in the context of rAAV.

**Table 3:** Immune suppression drugs currently used in gene therapy trials or under investigation in animal models

Drug	Mechanism of action	Also used in
Steroids	Binds glucocorticoid receptor, inhibits vasodilation, downregulates inflammatory gene expression	Inflammatory diseases
Rituximab	Deplete B cells	B cell lymphoma
CD19-specific CAR-T	Deplete B cells	B cell lymphoma
Rapamycin/ImmTOR	Inhibits mTOR	
Tacrolimus	Inhibits Calcinerin	Organ transplant
Anakinra	IL1R antagonist	RA
Azathioprine	Inhibits purine synthesis	RA, lupus, Inflammatory bowel diseases, transplant
Nipocalimab	Binds FcRn	HDFN
Daratumumab	Anti-CD38	MM
Bortezomib	Inhibits 26S proteosome	MM, RA
Imlifidase (IdeS)	Cuts IgG	Kidney transplant
Soluble CTLA-4	Binds CD80/86	
Clodronate	Depletes macrophages	Osteoporosis, pain control
Hydrogel	Slow release	
Extracellular vesicles	Coats AAV	
Dasatinib/Acalabrutinib	Kinase inhibitors	Leukemia

*ImmTOR* tolerogenic nanoparticles encapsulating rapamycin, *mTOR* mammalian target of rapamycin, *RA* rheumatoid arthritis, *HDFN* hemolytic disease of the fetus and newborn, *MM* multiple myeloma, *CTLA-4* cytotoxic T-lymphocyte–associated antigen 4

Three key areas require attention in advancing our understanding of rAAV immunology. First, there is a notable gap in studies examining the basic immunology of wtAAV, largely related to the fact that until very recently, wtAAV was not known to cause disease.^31,33^ Second, immunology research tends to focus on primary immune cells, cell lines derived from immune cells, or tumor cells. This overlooks the diverse somatic cells targeted by rAAV gene therapy in vivo (e.g., CNS cells, retinal cells, hepatocytes, muscle cells), each possessing different immunological features. Third, insights gained from animal models may not always reflect the human immune responses observed in clinical trials. Addressing these challenges could involve improved in vitro modeling systems such as mixed cell cultures, 3D organoids, and induced pluripotent stem cells.^454^ Additionally, the advancement of CRISPR screening technologies may help reveal cellular factors that participate in rAAV-based gene delivery.^207^ Innovative in vivo systems are also being developed, such as mice-engrafted human bone marrow, liver, and thymus (BLT) systems, which may be helpful for monitoring the activation of human innate sensors and the development of human adaptive immunity.^455^

### **Adverse effects/toxicities and management strategies**

Safety remains a major concern for therapy development. While rAAV is favored among currently available viral vectors because of its safety profile, it is not without risks. This section focuses on the side effects of rAAV that have clinically drawn attention (Table 4).

**Table 4:** Adverse effects and toxicities resulting from rAAV administration

Toxicity	Pathology	Proposed mechanism	Supportive Treatment	Possible Association
Genotoxicity	Tumorigenesis identified in animals but unknown in humans	DNA damage	Unknown	rAAV serotype, promoter/enhancer elements, vector purities
Hepatotoxicity	• Liver enzyme elevation	• DNA damage	Corticosteroids	i.v., dose
	• Drug-induced liver injury (rare)	• ER stress		
Thrombotic microangiopathy	• Endothelial vessel injury	Complement activation	• Corticosteroids	i.v., dose
	• Microvascular thrombosis		• Complement inhibition	
			• Plasmapheresis	
Dorsal root ganglion	• Immune cell infiltrates	Cellular overload with transgene	Unknown	c.m., i.t., dose
	• Nerve cell body degeneration			
Brain MRI findings	• Edema/inflammation	Unknown	Unknown	i.t., i.p., clinical significance unclear
	• Gliosis			
	• Immune cell infiltrates			

*ER* endoplasmic reticulum, *i.v.* intravenous, *c.m.* cisterna magna, *i.t.* intrathecal, *i.p.* intraparenchymal

#### Genotoxicity (insertional mutagenesis)

wtAAV can integrate into the mammalian genome, facilitated by elements such as Rep protein.^456^ However, ITRs are the only wtAAV elements present in rAAV, which should reduce the risk for genome integration. However, data in animal models suggest that the chance for viral genome integration is similar between wtAAV and rAAV. The liver appears to be a hotspot for AAV integration, but viral genome integration in other organs such as the heart has been found as well.^457^ Genomic integration is not detrimental unless it disrupts tumor-suppressing or activates oncogenic genes. The liver is highly transduced, particularly when AAV is delivered by i.v. administration. Thus, hepatocellular carcinoma (HCC) is of particular concern and has been widely described in animal studies.^457–465^ Interestingly, predisposing factors, such as animal age or underlying liver disease, correlate to the occurrence of HCC in animals. In contrast, no genotoxicity in NHPs or dogs has been described.^459^ Although wtAAV genome integration has been found in humans, a causative role in tumor development has not been established.^466–468^ Similarly, the role of therapeutic rAAV in human genome integration and its ability to cause cancer is unclear.^469^

The growing application of genome editing tools, particularly CRISPR, has led to an observed increase in rAAV genome integration following CRISPR-induced double-strand breaks in animal models, posing another challenge to safety.^470,471^ Other factors postulated to influence vector genome integration include rAAV serotype, promoter/enhancer elements, and vector impurities. Overall, the possibility and risk of therapeutic genome integration and its role in tumor formation in human patients are not known.

#### Hepatotoxicity

The administration of high doses of rAAV via i.v. injection is a strategy to achieve widespread transduction of different organs (e.g., muscle and CNS). However, this approach can cause liver toxicity as substantial numbers of viral particles pass through the liver upon entering circulation. Early signs of hepatotoxicity are elevated liver enzymes as monitored by blood testing, which can remain clinically silent or progress to signs of liver injury, failure, and even death.^265,472,473^ Prophylactic and post-dosing increase of corticosteroids has shown effects to mitigate hepatotoxicity. Liver toxicity after i.v. administration often occurs several weeks to months after treatment, suggesting that an immune response is responsible for the effect.^265,306,307,472^ This is also supported by earlier reports in hemophilia B, where decreased transgene expression was associated with T cell response against the rAAV capsid and rising liver enzymes.^306,474^

#### TMA

TMA is thought to result from endothelial injury with concomitant platelet aggregation, causing the formation of microthrombi, thrombocytopenia, end organ damage through ischemia, and possible death. It can be acquired or genetic^475,476^ and its association with rAAV gene therapy has only recently become apparent. Although the mechanism is not fully understood, dysregulated complement activation following rAAV infusion has been postulated. In addition to close patient monitoring, current strategies focus on prophylactic immune suppression. Successful treatment of TMA has been reported with supportive care, corticosteroids, eculizumab, and plasmapheresis.^412^ Most cases of rAAV-associated TMA have been reported for Zolgensma and in DMD trials.^412,413,477^ It is not known what factors could cause the predisposition to TMA after rAAV administration. Possible patient-specific factors such as the disease phenotype and the course of the underlying disease are starting to emerge.^413^

#### Neurotoxicity

##### DRG toxicity

DRG toxicity causing inflammation and neuronal loss in sensory ganglions with possible extension into dorsal spinal cord columns is a recently noted rAAV-associated side-effect.^442,478–481^ Current data are primarily from preclinical studies in rodents, monkeys, and piglets where i.v. and i.t. infusion of rAAV was associated with inflammatory infiltrates and neuronal degeneration. DRG toxicity can be preclinically silent, but studies described animal proprioceptive deficits and ataxia.^480^ Histopathological and clinical changes usually occur but can be delayed, suggesting that toxicity is related to the expression of the transgene rather than the capsid. In support of this, one study reported that no DRG toxicity was observed after the administration of a non-expressing rAAV9 vector.^479^ Recently, serum neurofilament (NF) levels were proposed as a marker,^442^ although how serum NF changes are clinically relevant to DRG toxicity is not known. In addition, serum NF changes might be disease-associated and part of the natural history, which would complicate the interpretation of NF levels.

Another challenge is the limited information about DRG toxicity in human patients. In a trial consisting of two patients given i.t. infusion of rAAV9 at a dose of 4.2 × 10^14^ vgs, one patient developed tingling in his arms and painful shooting pain in the left foot, which correlated with electrophysiologic changes and DRG contrast enhancement on MRI. The second patient received higher levels of immune suppression than the first patient and did not develop clinical signs of DRG toxicity.^445^ With an increasing number of patients receiving rAAV, the mechanisms, diagnosis, prevention, and treatment of neurotoxicities must be elucidated to improve safety and patient outcomes.

##### MRI findings

As medical technologies evolve, increased sensitivity facilitates the identification of cellular changes that were previously undetectable. This creates new challenges in how to interpret these findings and determine whether they are of clinical relevance. One example is changes in brain MRI detected after intraparenchymal injections of rAAV.

In preclinical studies, direct intraparenchymal rAAV delivery was associated with lymphocyte aggregation in perivascular spaces (lymphoplasmacytic perivascular cuffing), microglial changes, and neuronal swelling, in the area of the vector administration.^482–484^ Interestingly, changes were also found in the vehicle control group in one study. However, escalating doses resulted in a proportional increase in regions with T and B cell infiltrates, suggesting that changes are caused by the procedure and the vector.^484^ Although MRI changes were identified in both the vehicle and vector groups, the features might indicate different etiologies.^484^ Of note, no clinical changes were observed in this study.

In a human trial for Batten disease, rAAV.rh10 administration expressing *CLN2* was associated with post-infusion MRI changes, which continued to be present in most subjects by 12 months post-infusion. Importantly, no apparent clinical signs were attributed to those MRI findings.^485^ The significance of post-infusion MRI changes is currently unclear, particularly in neurodegenerative disease, where it might be challenging to discern disease-related changes from the procedure and vector-associated effects. However, understanding the underlying molecular and cellular mechanisms will be critical for patient safety and gene therapy success.

## Current gaps in knowledge and potential solutions

### Persistence

The promise of gene therapy as a single treatment for lifelong therapeutic effects depends on rAAV’s persistence. Though the immunity-mediated loss of rAAV genome or expressed transgene is excluded here (discussed in the section on rAAV immune response), two factors related to rAAV and patient remain regulatory elements of the expression cassette and cellular renewal/survival.

Endogenous genes are tightly regulated in a spatio-temporal manner, meaning that a specific gene is expressed at varying levels in different organs or even different cells within the same organ at a given time point. Widely used generic promoters are unable to accommodate such gene regulatory differences. Loss of transgene expression has been attributed to the deactivation of promoter and other regulatory elements used in the rAAV expression cassette design.^486^ In addition, promoters without cellular or organ specificity can present a challenge to safety. A possible solution is the use of promoter and regulatory elements derived from the endogenous promoter of the therapeutic transgene.^115^ However, endogenous regulatory elements typically exceed the packaging limit of rAAV and, therefore, require trimming, possibly excluding essential elements. A promising example is the use of a shortened SMN promoter for the treatment of SMA, which showed improved efficacy and reduced toxicity in mice.^487^

One challenge of using a partial promoter could be fluctuations in transgene expression during the development of an organism or patient, which could be desired if it reflects the endogenous gene expression but could pose a therapeutic issue if it compromises the therapeutic effect. It is, therefore, critical to better understand the physiologic regulation of the therapeutic gene.

From a host’s perspective, the rAAV genome’s persistence and transgene expression depend on the cells’ half-life time. This can be highlighted in two examples: neurons and the liver. Neurons are considered terminally differentiated, meaning that transduced neurons harbor the rAAV genome throughout the cell’s lifetime. Because the number of neurons does not substantially increase during life, it is unlikely that the rAAV genome would be diluted or even lost due to cellular proliferation. However, a partially treated neurodegenerative disease might be prone to ongoing neuronal loss and would eventually lose the rAAV genome and, thus, transgene expression.

In contrast, the cellular behavior of the liver is dynamic. Hepatocytes can proliferate continuously, with individual hepatocytes living for approximately three years.^488^ This could profoundly dilute the rAAV genome and even lead to transgene expression loss with the subsequent decline in therapeutic effect. As for most diseases, these aspects are not fully understood, emphasizing the need to better understand the molecular and cellular diseases for successful and long-lasting gene therapy.

While current rAAV therapies in humans have demonstrated encouraging results and life-changing outcomes, it is important to recognize that gene therapy is a relatively young field. The substantial number of patients receiving rAAV gene therapy is a recent accomplishment. It may be premature to conclude on questions of therapeutic effect persistence and the need to manage diminishing therapeutic effects, possibly through strategies such as re-dosing or combination with cell therapy.

### Disease-specific needs

Our mechanistic understanding of rAAV transduction has greatly increased over the years. However, whether and how molecular and cellular changes in different pathologies affect rAAV’s performance is poorly understood. For example, disease conditions could change the cellular lineage to affect the population of target cells. An additional complication is that similar dynamics might exist for regulatory elements that control transgene expression.

A goal of gene therapies is to benefit every patient, irrespective of their disease stage and progression. Patient trial eligibility is based on clinical, biochemical, and radiographic assessment. This approach can only approximate the disease stage but has limitations in predicting disease progression or response to treatment. While current data in humans highlight that earlier treatment correlates with better outcomes, the limitations in defining disease stage and correlating it to progression and response to gene therapy potentially exclude many patients who could otherwise have a favorable outcome. For example, two different hypotheses were postulated to explain the fatal outcome in two independent rAAV trials for DMD.^336^^,337^ This suggests that the ability to understand disease dynamics, particularly in the context of gene therapy administration, is still limited.

Overcoming these challenges is necessary to continue making advancements in the understanding of disease mechanisms and improvements of rAAV. For example, widespread targeting of muscle tissue for DMD is only likely to be achieved with i.v. delivery. Modifying rAAV capsids to enhance specificity and reduce dose will be crucial for improved safety and efficacy using this delivery method. Although isolated limb perfusion could serve as an alternative, it is unlikely sufficient to reach the entirety of disease pathology. However, combinations of routes of delivery might help to exploit the advantages and simultaneously reduce the disadvantages.

Another example is widespread CNS targeting. Although new routes of administration to confine rAAV to the CNS space (i.e., i.t., i.c.v.) are being tested, the widespread transduction of the CNS seems to benefit from the finely arborized blood vessel supply of the CNS, which is utilized by i.v. rAAV delivery. However, newer capsids might facilitate parenchymal penetration and intraparenchymal spread, thus reducing the disadvantages of i.v. delivery while achieving widespread cellular transduction.

## Conclusion

In conclusion, we have discussed the current understanding of AAV biology and manufacturing with a focus on the clinical trials of rAAV-based gene therapy and the challenges facing rAAV when applied to various diseases. The field is currently undergoing significant advancements, marked by progress in both academia and industry. Early successes in treating monogenic diseases have demonstrated the safety and effectiveness of rAAV-based gene therapy. However, challenges persist that must be addressed for a broader range of diseases. One prominent challenge is the effective delivery of rAAV to target tissues, where the choice of administration route can greatly impact the success of therapy. Addressing concerns related to specific cell/tissue targeting and overcoming physical barriers will be essential for expanding the range of targeted diseases. The immunogenicity of rAAV poses another challenge, impacting the safety, durability, and efficacy of gene therapy. Strategies to modulate and mitigate immune responses, including pre-existing immunity in patients, innate immune reactions upon vector administration, viral capsid, and transgene-induced adaptive immune response are essential for patient safety. Understanding the long-term consequences of immune reactions and their potential impact on repeated dosing will be critical. Finally, a better understanding of rAAV integration will inform its potential implications for tumor formation, hepatotoxicity, neurotoxicity, and newly detected adverse effects associated with high doses of rAAV need to be addressed to improve safety and accessibility for patients to this promising area of therapies.

Despite these challenges, several encouraging directions can advance the development of rAAV gene therapy. Research into rAAV-host interactions is vital for informing vector engineering and enhancing transgene expression. Strategies to achieve prolonged transgene expression, including immune evasion or immunosuppressive regimens, can extend the therapeutic benefits and reduce the need for repeated interventions. Tailoring rAAV-based therapies to disease-specific needs, such as customizing tissue, cell- and gene-specific promoters, optimizing transgene design, and engineering capsid variants with specific tissue tropism, represents another exciting direction. These newly engineered rAAVs must exhibit compatibility across species while demonstrating enhanced attributes and safety. Integrating rAAV with CRISPR-based tools, as gene editing technologies advance, offers remarkable potential for precise and targeted genetic modifications. These advancements promise to accelerate more effective treatments across a spectrum of diseases, from monogenic disorders to complex, multifactorial conditions. Overcoming current challenges and embracing these future directions holds the promise of turning gene therapy into a revolutionary power to address numerous human diseases.

### Supplementary information

Current clinical trials employing rAAV-based gene therapy for human diseases
